# Exosomal miR‐532‐5p induced by long‐term exercise rescues blood–brain barrier function in 5XFAD mice via downregulation of *EPHA4*

**DOI:** 10.1111/acel.13748

**Published:** 2022-12-09

**Authors:** Xiaoyan Liang, Wenxin Fa, Nan Wang, Yuanming Peng, Cuicui Liu, Min Zhu, Na Tian, Yongxiang Wang, Xiaolei Han, Chengxuan Qiu, Tingting Hou, Yifeng Du

**Affiliations:** ^1^ Department of Neurology Shandong Provincial Hospital, Shandong University Jinan Shandong China; ^2^ Department of Neurology Shandong Provincial Hospital Affiliated to Shandong First Medical University Jinan Shandong China; ^3^ Department of Clinical Laboratory Third Hospital of Jinan Shandong China; ^4^ Shandong Provincial Clinical Research Center for Neurological Diseases Jinan Shandong China; ^5^ Aging Research Center and Center for Alzheimer Research, Department of Neurobiology, Care Sciences and Society Karolinska Institutet‐Stockholm University Solna Sweden

**Keywords:** Alzheimer's disease, blood–brain barrier, exercise, exosome, miR‐532‐5p

## Abstract

The breakdown of the blood–brain barrier, which develops early in Alzheimer's disease (AD), contributes to cognitive impairment. Exercise not only reduces the risk factors for AD but also confers direct protection against cognitive decline. However, the exact molecular mechanisms remain elusive, particularly whether exercise can liberate the function of the blood–brain barrier. Here, we demonstrate that long‐term exercise promotes the clearance of brain amyloid‐β by improving the function of the blood–brain barrier in 5XFAD mice. Significantly, treating primary brain pericytes or endothelial cells with exosomes isolated from the brain of exercised 5XFAD mice improves cell proliferation and upregulates PDGFRβ, ZO‐1, and claudin‐5. Moreover, exosomes isolated from exercised mice exhibit significant changes in miR‐532‐5p. Administration or transfection of miR‐532‐5p to sedentary mice or primary brain pericytes and endothelial cells reproduces the improvement of blood–brain barrier function. Exosomal miR‐532‐5p targets *EPHA4*, and accordingly, expression of EphA4 is decreased in exercised mice and miR‐532‐5p overexpressed mice. A specific siRNA targeting *EPHA4* recapitulates the effects on blood–brain barrier‐associated cells observed in exercised 5XFAD mice. Overall, our findings suggest that exosomes released by the brain contain a specific miRNA that is altered by exercise and has an impact on blood–brain barrier function in AD.

## Graphical abstract

Cells improves the function of the blood‐brain barrier, promoting the clearance of Aß across the blood‐brain barrier.

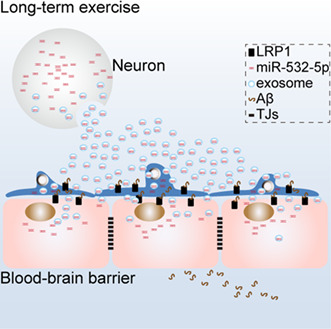

**Abbreviations**
AAV: adeno‐associated virus vector
Aβ: amyloid‐β
AβO: Aβ oligomer
AD: Alzheimer’s disease
BBB: blood‐brain barrier
CBF: cerebral blood flow
CNS: central nerve system
EB: Evans Blue dye
EphA4: Eph receptor A4
EXE‐5XFAD: exercised 5XFAD
EXE‐exo: the exosomes of EXE‐5XFADmice
EXE‐WT: exercised wild‐type
MWM: Morris Water Maze
NC: negative control
SED‐5XFAD: sedentary 5XFAD
SED‐exo: the exosomes of SED‐5XFAD mice
SED‐WT: sedentarywild‐type
siRNA: small interfering RNA
TJs: tight junctions

## 1 INTRODUCTION

Alzheimer's disease (AD), the most common cause of dementia, is a progressive neurodegenerative disorder that is clinically evident with memory deficits (Lane et al., 2018). In addition to the amyloid hypothesis (Paroni et al., 2019), the involvement of cerebrovascular dysfunction, especially blood–brain barrier (BBB) impairments, in the progression of AD has been increasingly appreciated (Cai et al., 2018). Changes in BBB are an early preclinical feature of AD pathology with reductions in cerebral blood flow (CBF) (Lacalle‐Aurioles et al., 2014), increases in BBB permeability, loss of tight junctions (TJs), and impairment of pericytes (Nation et al., 2019; Shi et al., 2020). As the disease progresses, the breakdown of the BBB impedes the clearance of neurotoxic molecules like amyloid‐β (Aβ), resulting in cognitive impairment (Ma et al., 2018; Storck et al., 2016). Therefore, the BBB may represent a novel therapeutic target to modify disease progression in AD.

In addition to reducing conventional AD risk factors (Hou et al., 2019), exercise also provides direct endogenous neuroprotection, including preventing age‐related neurovascular decline. Previous studies have shown that exercise mitigates BBB dysfunction by restoring the expression of TJs and preserving the function of pericytes in the aging process (Soto et al., 2015) and several chronic inflammatory diseases such as multiple sclerosis (Souza et al., 2017). However, whether exercise can ameliorate the BBB damage in AD pathology and its mechanism remain unclear. Recently, exosomes, the critical medium for cell‐to‐cell communication, have attracted intense attention in the context of exercise (Nair et al., 2020). An increasing number of studies have revealed that their release during exercise can act in an “endocrine manner” (Whitham et al., 2018) via mediating the horizontal transfer of biologically active molecules, including proteins, lipids, mRNA, and non‐coding RNAs (e.g., miRNAs), thereby impacting the recipient cell function. Out of them, miRNAs, which downregulate the function of the target gene, can actively respond to exercise (Nair et al., 2020) and significantly modulate several disease processes, including cognition recovery in post‐stroke (Ge et al., 2020), brain vascular integrity (Xu et al., 2017), and early brain injury (Chen et al., 2020).

Hence, in the present study, we sought to determine whether exosomal miRNAs induced by exercise may convey part of the beneficial effects on memory by modifying the function of the BBB in 5XFAD mice. Our study demonstrates that exercise upregulates a specific miRNA level in brain exosomes, which induces function alleviation in the BBB, thus eventually reducing the brain Aβ load and improving memory abilities in 5XFAD mice.

## 2 RESULTS

### 2.1 Long‐term exercise improves brain Aβ clearance across the BBB and ameliorates memory impairment in 5XFAD mice

**FIGURE 1:**
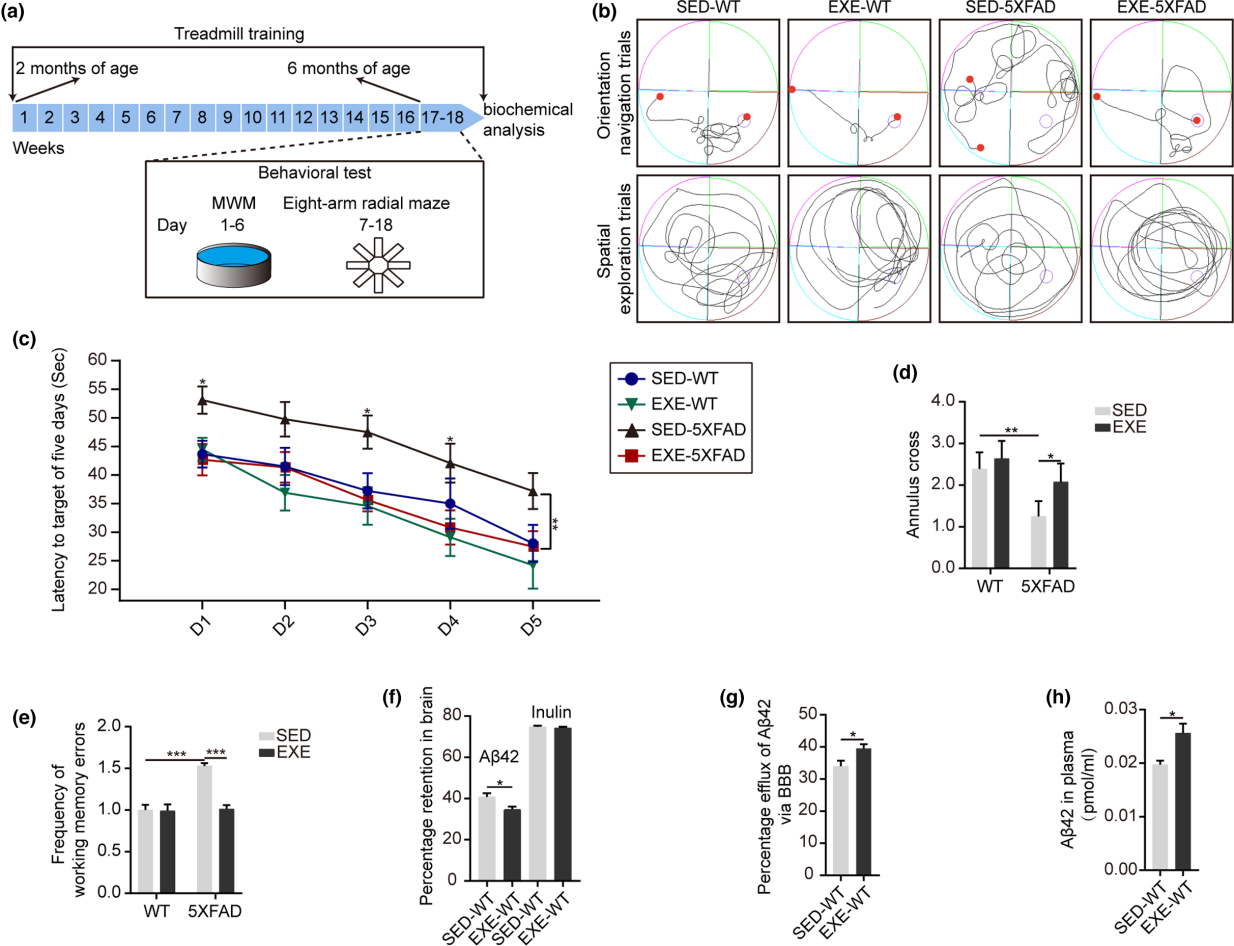
Long‐term exercise improves brain Aβ clearance across the BBB and alleviates memory abilities in 5XFAD mice. (a) Schematic illustration of the study design and workflow. (b) Representative images of the route track in the orientation navigation trials and spatial exploration trials. (c) Latency curves showing the time trend to reach the hidden platform in 5 days. *N* = 19 mice per group; Mean ± SEM; *p < 0.05, **p < 0.01. (d) Numbers of platform crossings tested in the spatial exploration trials. *N* = 19 mice per group; Mean ± SEM; *p < 0.05, **p < 0.01 by two‐way ANOVA. (e) Comparison of the frequency of working memory errors during the learning session. *N* = 8 mice per group; Mean ± SEM; ***p < 0.001 by two‐way ANOVA. (f–h) Comparison of the brain retention of Aβ42 (left) and inulin (right) (f), Aβ42 clearance across the BBB (g), and plasma level of Aβ42 (h) after 30 min of intracerebral administration of human Aβ42 and inulin into the caudate nucleus between SED‐WT mice and EXE‐WT mice. *N* = 3 mice per group; Mean ± SEM; *p < 0.05 by two‐tailed *t* test.

After 16 weeks of treadmill training (Figure 1a), we evaluated the memory ability of mice using the Morris Water Maze (MWM) and an eight‐arm radial maze. We observed that the route track of sedentary 5XFAD (SED‐5XFAD) mice was marginal style, while the route track of exercised 5XFAD (EXE‐5XFAD) mice was taxis style in orientation navigation trials (Figure 1b), suggesting that EXE‐5XFAD mice have more exploration awareness. Meanwhile, the escape latency time of EXE‐5XFAD mice was significantly shorter, compared with SED‐5XFAD mice, by 18% (Figure 1c and Figure S1b). In the spatial exploration experiment, results showed that the route track of EXE‐5XFAD mice was targeted (Figure 1b) and the numbers of platform crosses by EXE‐5XFAD mice were significantly higher than those by SED‐FAD mice (Figure 1d). In addition to spatial memory, we also observed a significantly low frequency of working memory and reference memory errors in EXE‐5XFAD mice when compared to SED‐5XFAD mice (Figure 1e and Figure S1c,d). There was no significant difference in swimming speed between sedentary mice and exercised mice (Figure S1a) and no apparent differences in memory performance between sedentary wild‐type (SED‐WT) mice and exercised wild‐type (EXE‐WT) mice.

To examine whether exercise plays a role in Aβ clearance across the BBB in vivo, we studied clearance of human Aβ42 in WT (sedentary and exercised) mice. Using an Aβ clearance assay (Zhao et al., 2015) and ELISA analysis, we revealed that EXE‐WT mice have lower brain retention of Aβ42 30 min following intracerebral administration of human Aβ42 when compared to SED‐WT mice (Figure 1f). No difference in the retention of inulin, an inert extracellular space marker, was observed. In line with these results, exercise increased Aβ42 efflux across the BBB by 14% compared with controls (Figure 1g). In addition, we also found a greater plasma level of Aβ42 in EXE‐WT mice compared with SED‐WT mice, confirming improved Aβ clearance from the brain to blood (Figure 1h).

### 2.2 Long‐term exercise improves the structure and function of the BBB in 5XFAD mice

**FIGURE 2:**
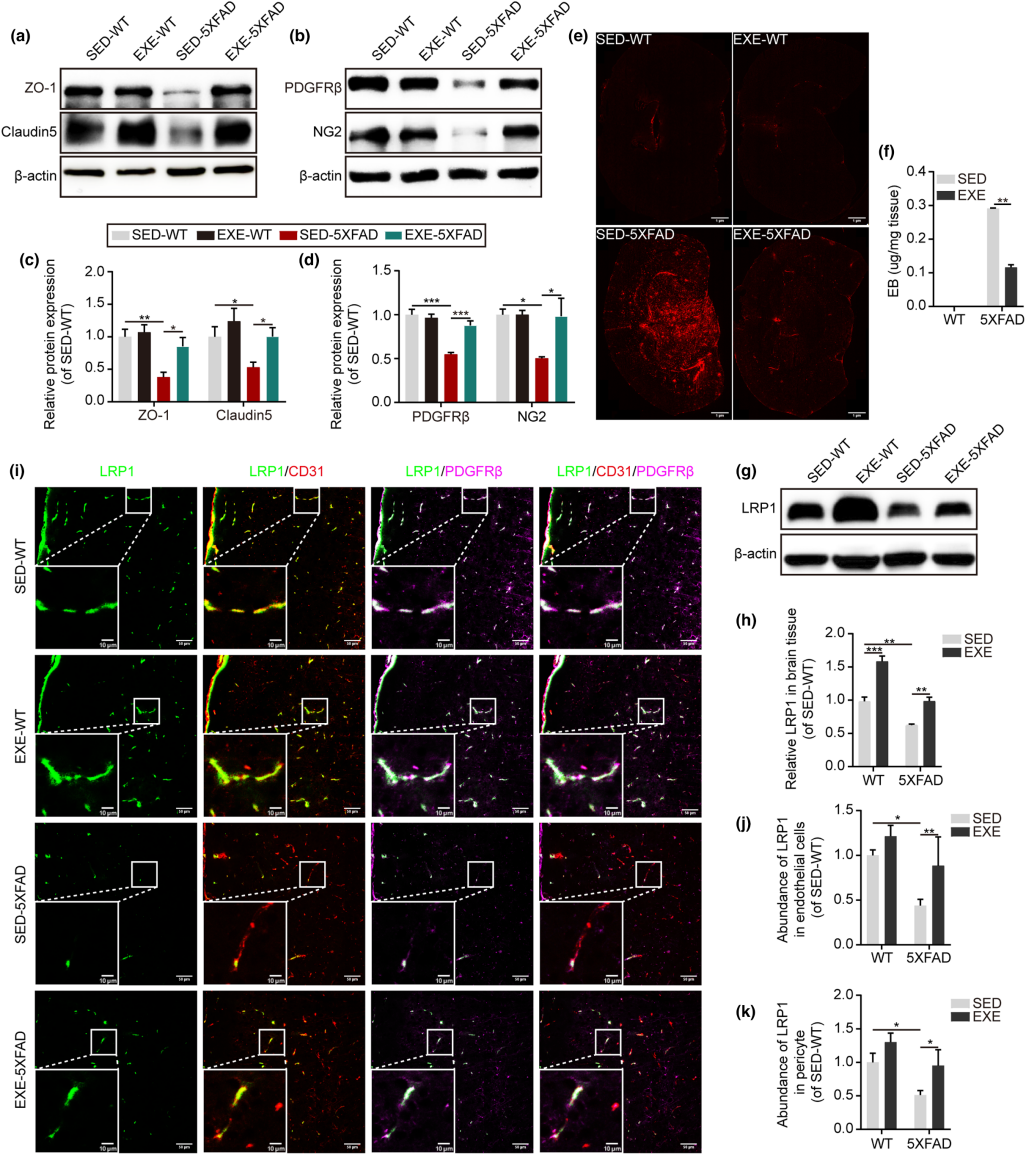
Long‐term exercise improves the structure and function of BBB in 5XFAD mice. (a) Immunoblotting of ZO‐1 and claudin‐5 in isolated brain tissue from 6‐month‐old SED‐WT, EXE‐WT, SED‐5XFAD, and EXE‐5XFAD mice. (b) Immunoblotting of PDGFRβ and NG2 in isolated brain tissue from 6‐month‐old SED‐WT, EXE‐WT, SED‐5XFAD, and EXE‐5XFAD mice. (c) Quantification of ZO‐1 and claudin‐5 relative expression by grey values analysis of immunoblots in WT and 5XFAD mice. β‐actin was used as a control. *N* = 7 mice per group; Mean ± SEM; *p < 0.05, **p < 0.01 by two‐way ANOVA. (d) Quantification of PDGFRβ and NG2 relative expression by grey values analysis of immunoblots in WT and 5XFAD mice. β‐actin was used as a control. *N* = 7 mice per group; Mean ± SEM; *p < 0.05, **p < 0.01, ***p < 0.001 by two‐way ANOVA. (e) Permeability of brain micro‐vessels studied by microscopy analysis of Evans Blue dye in 6‐month‐old SED‐WT, EXE‐WT, SED‐5XFAD, and EXE‐5XFAD mice. Scan bar: 1 μm. (f) Quantification of Evans Blue dye leakage in the whole brain by *UV* spectrophotometer analysis in WT and 5XFAD mice. *N* = 3 mice per group; Mean ± SEM; **p < 0.01 by two‐tailed *t* test. (g) Immunoblotting of LRP1 in isolated brain tissue from 6‐month‐old SED‐WT, EXE‐WT, SED‐5XFAD, and EXE‐5XFAD mice. (h) Quantification of LRP1 relative expression by grey values analysis of immunoblots in WT and 5XFAD mice showing an increased expression in exercised mice (EXE‐WT and EXE‐5XFAD). β‐actin was used as a control. *N* = 7 mice per group; Mean ± SEM; **p < 0.01, ***p < 0.001 by two‐way ANOVA. (i) Representative confocal microscopy images showing LRP1 (green) colocalization with PDGFRβ^+^ pericyte (magenta) and CD31^+^ endothelial cells (red) in brain cortical sections from 6‐month‐old SED‐WT, EXE‐WT, SED‐5XFAD, and EXE‐5XFAD mice. Scan bar: 50 μm for large images and 10 μm for the inset. (j and k) Quantification of an abundance of LRP1 on pericytes (j) and endothelial cells (k) respectively by fluorescence intensity analysis. *N* = 5 mice per group; Mean ± SEM; *p < 0.05, **p < 0.01 by two‐way ANOVA.

The maintenance of the structure and function of BBB is the basis of Aβ clearance across BBB (Pflanzner et al., 2011). As a result, we investigated whether long‐term exercise could improve BBB structure in 5XFAD mice. Western blot and immunostaining analysis of TJ proteins in the brain tissue revealed reduced expression of ZO‐1 and claudin‐5 in SED‐5XFAD mice compared with SED‐WT mice. However, after long‐term exercise, protein expression of ZO‐1 and claudin‐5 in 5XFAD mice was significantly elevated (2.2‐fold and 1.89‐fold respectively), indicating the positive effect of exercise on TJ proteins expression (Figure 2a,c, Figure S2a–c). Then, we investigated the expression of pericytes‐associated proteins including PDGFRβ and NG2. As shown in Figure 2b,d, SED‐5XFAD mice exhibited a significant pericyte deficiency with 45% and 50% reductions in PDGFRβ and NG2 expression, respectively, compared with SED‐WT mice. After long‐term exercise, EXE‐5XFAD mice showed approximately 60% and 95% increases in PDGFRβ and NG2 expression, respectively. These findings were also supported by immunostaining analysis for PDGFRβ‐positive pericytes (Figure S2a,d). Meanwhile, the BBB permeability assay revealed a 60% decreased BBB permeability to Evans Blue dye in EXE‐5XFAD mice, compared with SED‐5XFAD mice, further confirming improved BBB structure after exercise (Figure 2e,f). We failed to detect a significant variation in cerebral blood flow between groups (Figure S2f,g).

LRP1 downregulation in endothelial cells and pericytes was identified as a predominant event compromising the clearance of Aβ in AD patients and AD animal models (Storck et al., 2016). Thus, we further analyzed the abundance of LRP1 in the whole brain and key components of the BBB, respectively. Our results showed that the expression of LRP1 in SED‐5XFAD mice was reduced by ~36% compared with SED‐WT mice. After long‐term exercise, the expression of LRP1 increased significantly in both EXE‐WT mice and EXE‐5XFAD mice (Figure 2g,h, Figure S2a,e). Furthermore, a substantial reduction in LRP1 in endothelial cells and pericytes levels was found in our study, by approximately 56% and 49%, respectively, consistent with a reduction in AD patients (Halliday et al., 2016). Notably, after exercise, LRP1 in endothelial cells and pericytes of EXE‐5XFAD mice showed approximately 1.86‐fold and 2‐fold increases, respectively, compared with SED‐5XFAD mice, as illustrated by representative microscopy images (Figure 2i–k).

### 2.3 Brain exosomes from EXE‐5XFAD mice improve the function of BBB‐associated cells in vitro

**FIGURE 3:**
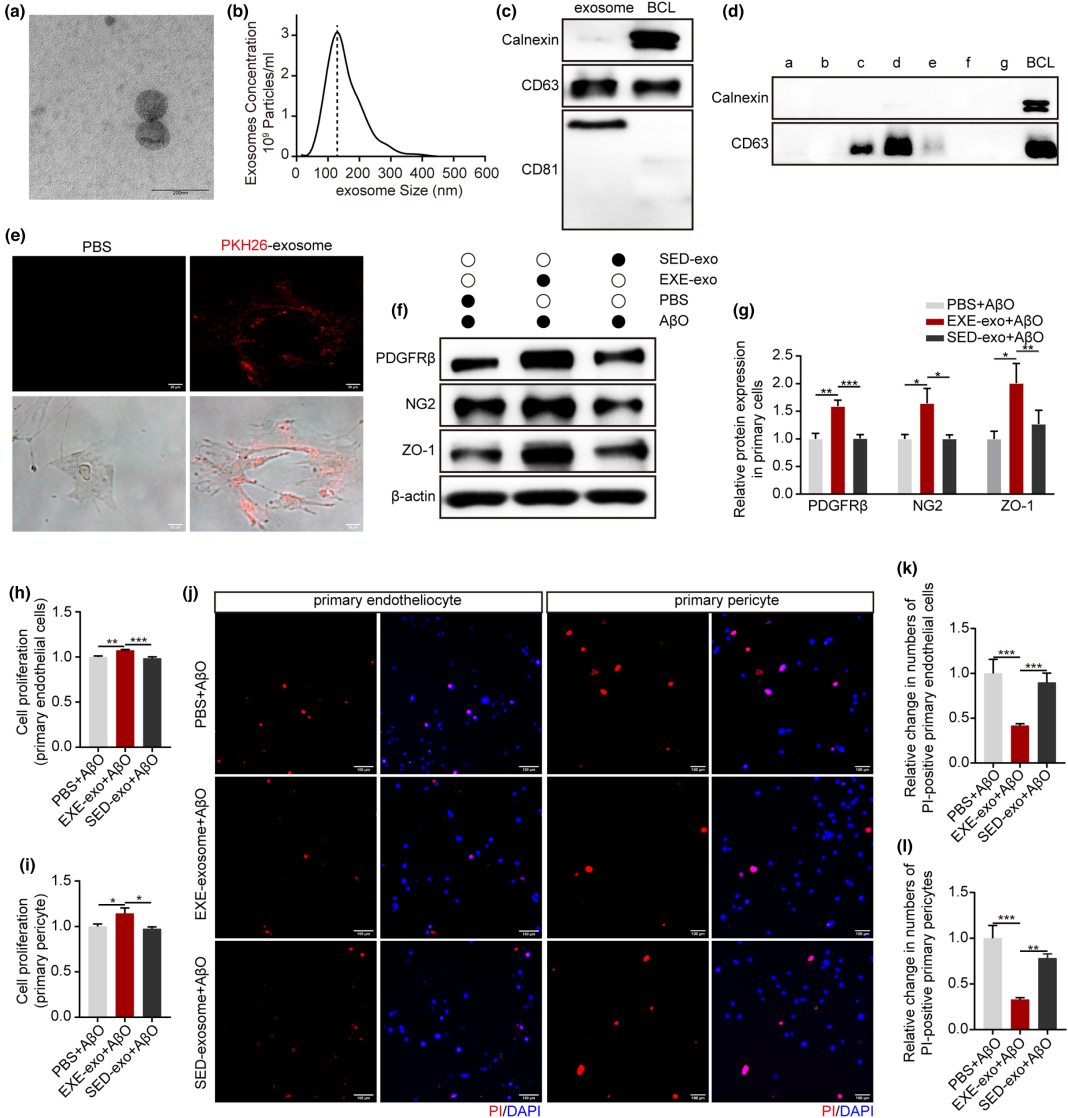
Characterization and functional validation of brain exosomes from EXE‐5XFAD mice. (a) Representative electron micrograph of isolated brain exosomes. Scale bar: 200 nm. (b) Representative results of nanoparticle tracking analysis demonstrating size distribution of brain exosomes. (c) Representative blots of brain exosomes marker proteins CD63 and CD81 and endoplasmic reticulum protein Calnexin showing vesicles purified using differential centrifugation were exosomes. (d) Representative blots of brain exosomes marker proteins CD63 and endoplasmic reticulum protein Calnexin showing exosomes purified using sucrose density gradient centrifugation existed mainly in c–e fractions. (e) Primary pericytes were cultured in the presence or absence of PKH26‐labeled exosomes (red) at 37°C for 6 h. Scan bar: 20 μm. (f) Immunoblotting of PDGFRβ, NG2, and ZO‐1 in cultured primary pericytes and endothelial cells, respectively, treated with PBS, SEX‐exo, and EXE‐exo. (g) Quantification of PDGFRβ, NG2, and ZO‐1 relative expression in primary pericytes and endothelial cells treated with PBS, SEX‐exo, and EXE‐exo respectively. β‐actin was used as a control. *N* = 3 replicates; Mean ± SEM; *p < 0.05, **p < 0.01, ***p < 0.001 by one‐way ANOVA. (h and i) MTT assay showed that exosomes isolated from the brain of EXE‐5XFAD mice improved cell proliferation both in endothelial cells (h) and pericytes (i). *N* = 3 replicates; Mean ± SEM; *p < 0.05, **p < 0.01, ***p < 0.001 by one‐way ANOVA. (j) Primary pericytes and endothelial cells were treated with PBS, SED‐exo and EXE‐exo for 48 h. Representative micrograph of cell apoptosis determined by PI staining (red). Scan bar: 100 μm. (k, l) Quantification of the apoptosis of primary endothelial cells (k) and pericytes (l). Cell apoptosis was determined as a percentage (%) of PI‐positive cells covering all DAPI‐positive cells. *N* = 3 replicates; Mean ± SEM; **p < 0.01, ***p < 0.001 by one‐way ANOVA.

The release of exosomes from cells or tissues into the extracellular environment has been proposed as an essential mechanism of intercellular communication in response to exercise (Whitham et al., 2018). Here, we investigated whether long‐term exercise affects BBB function via exosomes. We isolated exosomes from equal amounts of brain tissue of the exercised mice and sedentary mice within 24 h after the last training session using a standard protocol (Perez‐Gonzalez et al., 2017). An electron microscope characterized vesicle morphology as typical rounded particles with diameters of 50–150 nm (Figure 3a). Size distribution was determined by NTA (Figure 3b). Western blot analysis showed that the isolated vesicles expressed well‐known exosomal markers such as CD63 and CD81, but did not express the endoplasmic reticulum protein calnexin (Figure 3c,d). All the data confirmed the exosome identity of the isolated vesicles. We then labeled the exosomes with a lipid‐associating fluorescent dye, PKH26, and incubated them with primary pericytes for 6 h. As shown in Figure 3e, cells co‐cultured with labeled exosomes exhibited clear fluorescent signals in the cytoplasm, while no fluorescence signal was observed in the control. This implied that exosomes were internalized by recipient cells. To further determine the effects of exosomes of EXE‐5XFAD mice (EXE‐exo) on recipient cells, we exposed primary pericytes and endothelial cells to AβO (3 μM) for 24 h to mimic the AD environment. Then, PBS, EXE‐exo, and exosomes of SED‐5XFAD mice (SED‐exo) were added respectively to the medium for 48 h. Western blot analysis of PDGFRβ, NG2, and ZO‐1 showed that their expression increased by 58%, 64%, and 58%, respectively, in the EXE‐exo+ AβO group, compared with the SED‐exo+ AβO group (Figure 3f,g). MTT assay analysis revealed that EXE‐exo increased cell proliferation both in pericytes and endothelial cells (Figure 3h,i). Compared with SED‐exo, EXE‐exo also decreased the AβO‐induced apoptosis of pericytes and endothelial cells (Figure 3j–l). However, no obvious differences in cell proliferation, cell apoptosis, and expression of PDGFRβ, NG2, and ZO‐1 were found between the PBS+ AβO group and the SED‐exo+ AβO group.

### 2.4 MiR‐532‐5p is identified as a potential regulator of EXE‐exo‐induced BBB protection in vivo and in vitro

**FIGURE 4:**
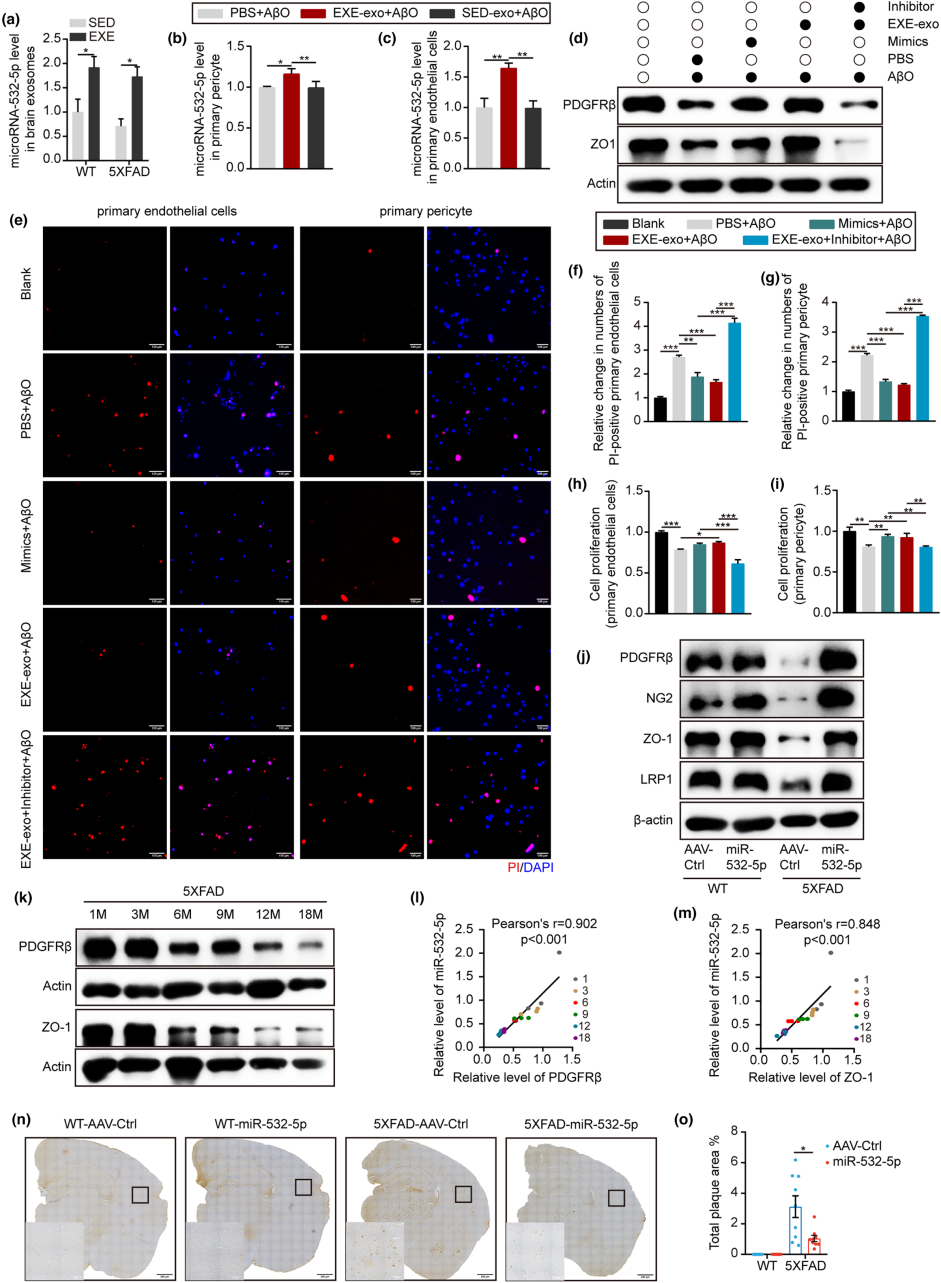
miR‐532‐5p was essential in the protection of the BBB. (a) Quantitative RT‐qPCR analysis of miR‐532‐5p in exosomes purified from the brain tissue of 6‐month‐old SED‐WT, EXE‐WT, SED‐5XFAD, and EXE‐5XFAD mice. Values are 2^−ΔΔt^. Data are normalized to U6; *N* = 6 mice per group; Mean ± SEM; *p < 0.05 by two‐way ANOVA. (b) Quantitative RT‐qPCR analysis of miR‐532‐5p in primary pericytes treated with PBS, SED‐exo and EXE‐exo for 48 h. Values are 2^−ΔΔt^. Data are normalized to U6; *N* = 3 replicates; Mean ± SEM; *p < 0.05, **p < 0.01 by one‐way ANOVA. (c) Quantitative RT‐qPCR analysis of miR‐532‐5p in primary endothelial cells treated with PBS, SED‐exo and EXE‐exo for 48 h. Values are 2^−ΔΔt^. Data are normalized to U6; *N* = 3 replicates; Mean ± SEM; **p < 0.01 by one‐way ANOVA. (d) Representative blots of PDGFRβ and ZO‐1 in primary cells transfected with a mimic or inhibitor of miR‐532‐5p. *N* = 3 replicates. (e) Representative micrograph of cell apoptosis determined by PI staining (red) in primary endothelial cells and pericytes transfected with a mimic or inhibitor of miR‐532‐5p for 48 h. Scan bar: 100 μm. (f and g) Quantification of primary endothelial cells (f) and pericytes (g) apoptosis. cell apoptosis was determined as a percentage (%) of PI‐positive cells covering all DAPI‐positive cells. *N* = 3 replicates; Mean ± SEM; **p < 0.01, ***p < 0.001 by one‐way ANOVA. (h and i) MTT assay analysis for cell proliferation in primary endothelial cells (h) and pericytes (i) transfected with a mimic or inhibitor of miR‐532‐5p for 48 h. *N* = 3 replicates; Mean ± SEM; *p < 0.05, **p < 0.01, ***p < 0.001 by one‐way ANOVA. (j) Representative blots of PDGFRβ, NG2, ZO‐1, and LRP1 in isolated brain tissue showing changes in protein expression after overexpression of miR‐532‐5p in vivo. *N* = 4 mice per group. (k) Representative blots of PDGFRβ and ZO‐1 in the brain of 5XFAD mice showing an age‐dependent loss of protein expression. *N* = 3 mice per group. (l and m) Positive correlation between age‐dependent reduction in PDGFRβ (l) and ZO‐1 (m) and miR‐532‐5p. *r* = Pearson's coefficient. *N* = 3 mice per group. (n) Representative microscopy analysis of 4G8 immunodetection in the brain of 5XFAD and WT mice (miR‐532‐5p overexpressed and control). Scan bar: 200 μm for large images and 50 μm for the inset. (o) Quantification of total plaque area in the whole brain. *N* = 3 mice per group; Mean ± SEM; *p < 0.05 by two‐tailed *t* test.

To investigate how EXE‐exo modified the BBB function in AD pathology, we first tested whether long‐term exercise influenced the levels of exosomes. Results from NTA showed no significant alterations in the size distribution or concentration of serum or brain exosomes between exercised mice and sedentary mice, suggesting that exosome cargoes, instead of amounts, might act as a mechanism to spread exercise‐induced changes across the BBB (Figure S3a–d). A growing number of studies have shown that exosomes regulate the protein expression of recipient cells and modify cell characteristics through miRNA transfer (Castano et al., 2020; Ge et al., 2020; Hou et al., 2019; Xu et al., 2017). Thus, we further explored the changes in exosomal miRNAs after exercise. In light of previous reports, we selected five candidates for miRNAs, including miR‐27‐3p (Harati et al., 2022), miR‐124‐3p (Ge et al., 2020; Tang et al., 2017), miR‐132‐3p (Xu et al., 2017), miR‐181‐5p (Kazenwadel et al., 2010), and miR‐532‐5p (Slater et al., 2018), for further screening via RT‐qPCR analysis, which was known to be involved in the regulation of vascular function. As the beneficial exosomes, regardless of their origin, all acted within the brain, we first screened these miRNAs within brain‐derived exosomes. Out of these five miRNAs, only miR‐532‐5p was found to be differentially expressed in brain‐derived exosomes between exercised mice and sedentary mice (Figure 4a, Figure S4a–d). Furthermore, when we treated primary pericytes and endothelial cells with EXE‐exo and SED‐exo, respectively, we found that the content of miR‐532‐5p in the EXE‐exo group was significantly higher than that in the SED‐exo group (Figure 4b,c). To test the function of miR‐532‐5p upon BBB, EXE‐exo with or without miR‐532‐5p inhibitor was added to the AβO‐pretreated cells for 48 hours. Cells co‐cultured with EXE‐exo were set as a positive control. Western Blot analysis showed that transfection of miR‐532‐5p mimics resulted in increased expression of PDGFRβ and ZO1, similar to the effects of EXE‐exo. Conversely, the expression of PDGFRβ and ZO1 in cells co‐cultured with EXE‐exo and miR‐532‐5p inhibitors was significantly reduced (Figure 4d, Figure S5a,b). Cell apoptosis and proliferation were also assessed. We obtained similar results in which miR‐532‐5p inhibitors reduced or even abrogated the positive effect of the EXE‐exo (Figure 4e–i). To provide further evidence that miR‐532‐5p plays an important role in BBB function, we overexpressed miR‐532‐5p in vivo. An adeno‐associated virus vector (AAV) was constructed and injected into the bilateral ventricle of 5‐month‐old mice via the stereotaxic technique to achieve a broad distribution of miR‐532‐5p in the brain. Five weeks after administering, we first evaluated the efficiency of the virus transfection. Represented immunofluorescence images showed that intracerebroventricular injections of AAV resulted in GFP expression through the whole brain (Figure S5c). Like exercise, overexpression of miR‐532‐5p in the brain of 5XFAD mice remarkably increased expression of PDGFRβ, NG2, ZO‐1, and LRP1 by 2.5‐fold, 3‐fold, 2.2‐fold, and 1.5‐fold respectively, when compared to controls (Figure 4j and Figure S5d). A slightly difference in the expression of these proteins was observed in WT mice. Moreover, we also explored the changes in miR‐532‐5p, PDGFRβ, and ZO‐1 with age and the correlation between miR‐532‐5p and PDGFRβ or ZO‐1 during aging. In 5XFAD mice, the analysis of RT‐qPCR indicated an age‐dependent reduction in the level of miR‐532‐5p, starting at 3 months of age (Figure S5e). Western blot analysis showed an age‐dependent loss of PDGFRβ starting from 3 to 6 months of age, with approximately 47% and 72% loss at 6 and 12 months of age, respectively (Figure 4k and Figure S5f). The loss of ZO‐1 displayed a similar decreasing trend as for PDGFRβ but started from 1 to 3 months of age, earlier than PDGFRβ (Figure 4k and Figure S5g). In WT mice, miR‐532‐5p content declined from 12 to 18 months of age, a later period than in 5XFAD mice (Figure S5h). PDGFRβ and ZO‐1 loss started at 6–9 months of age, also later than that in 5XFAD mice (Figure S5i–k). There was a strong positive correlation between an age‐dependent loss in PDGFRβ or ZO‐1 and age‐related changes in miR‐532‐5p, indicating that the changes in miR‐532‐5p with age in the brain were associated with the progressive age‐dependent BBB degeneration in 5XFAD mice (Figure 4l,m). We also found a weakly positive correlation between age‐related changes in PDGFRβ and miR‐532‐5p in the brain of WT mice, while no correlation was found between ZO‐1 and miR‐532‐5p in WT mice (Figure S5l,m). Finally, using immunostaining with 4G8 antibody, we examined the effect of miR‐532‐5p on Aβ load. Results revealed a decreased Aβ burden in miR‐532‐5p‐overexpressed 5XFAD mice compared with controls (Figure 4n,o).

### 2.5 Long‐term exercise promotes “cross‐talk” between neurons and the BBB via delivering miR‐532‐5p by exosomes

**FIGURE 5:**
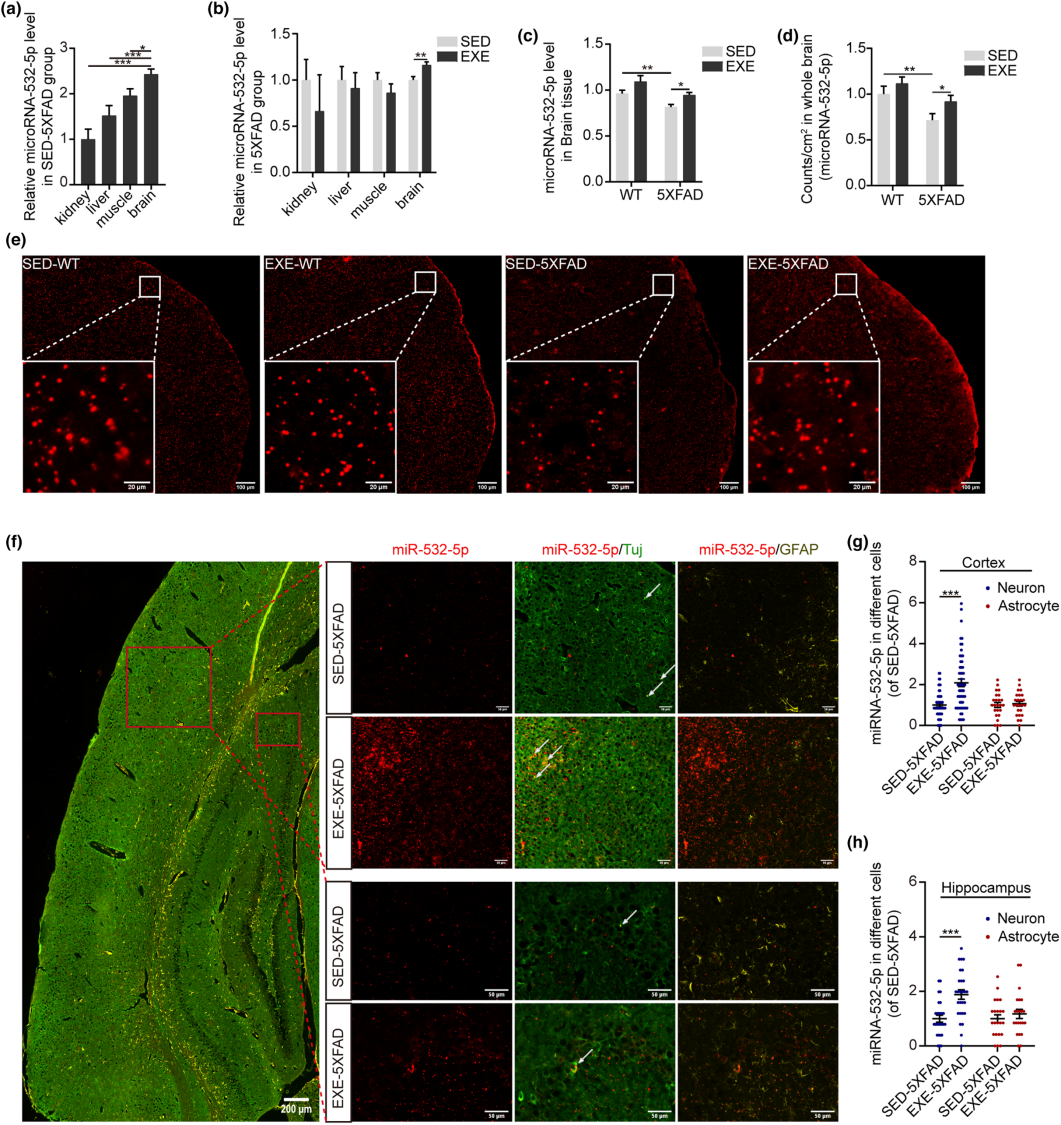
Long‐term exercise increased the expression of miR‐532‐5p in neurons. (a) Quantitative RT‐qPCR analysis of miR‐532‐5p in four tissues of 5XFAD mice expressed as fold change to the kidney. U6 was used as an internal control. *N* = 3; Mean ± SEM; **p < 0.01, ***p < 0.001 by one‐way ANOVA. (b) Quantitative RT‐qPCR analysis of miR‐532‐5p in four tissues from SED‐5XFAD and EXE‐5XFAD mice. Data were presented as fold change of SED‐5XFAD. U6 was used as an internal control. *N* = 3; Mean ± SEM; **p < 0.01 by one‐way ANOVA. (c) Quantitative RT‐qPCR analysis of miR‐532‐5p in brain tissue from SED‐WT, EXE‐WT, SED‐5XFAD, and EXE‐5XFAD mice. Data were presented as fold change of SED‐WT. U6 was used as an internal control. *N* = 3 mice per group; Mean ± SEM; *p < 0.05 by two‐way ANOVA. (d) Quantification of miR‐532‐5p relative level by counts analysis. *N* = 4 mice per group; Mean ± SEM; *p < 0.05, **p < 0.01 by two‐way ANOVA. (e) Representative images of miR‐532‐5p level in the whole brain by miRNA chromogenic in situ hybridization analysis. Scan bar: 100 μm for large images and 20 μm for the inset. (f) Representative confocal microscopy images showing miR‐532‐5p (red) colocalization with TUJ^+^ neurons (green) and GFAP^+^ astrocyte (yellow) in the cortex and hippocampus of 6‐month‐old 5XFAD mice. Scan bar: 200 μm for left images and 50 μm for the right. (g and h) Quantification of the level of miR‐532‐5p on neurons and astrocytes in the cortex (g) and hippocampus (h) of 5XFAD mice. *N* = 4 mice per group; Mean ± SEM; ***p < 0.001 by two‐way ANOVA.

Considering that peripheral tissues such as skeletal muscle (Castano et al., 2020) and liver (Hansen et al., 2011) have also been demonstrated to release functional exosomes during exercise, as well as the fact that peripheral exosomes can interact with the BBB, leading to changes in the barrier's properties (Saint‐Pol et al., 2020), an important question is to identify which tissue is the main source of these exosomal miR‐532‐5p induced by long‐term exercise. We examined the expression of miR‐532‐5p in the kidney, liver, muscle, and brain of mice. Collectively, miR‐532‐5p is expressed in multiple tissues but is most abundant in the brain compared with other tissues under sedentary status, suggesting that miR‐532‐5p more likely originates from the brain (Figure 5a and Figure S6a). In addition, it is in the brain rather than the kidney, liver, or muscle that exercise induced the most significant increase in miR‐532‐5p in 5XFAD mice, further supporting our conclusion that the brain is the major origin of miR‐532‐5p (Figure 5b,c). However, we did not observe significant differences in changes of miR‐532‐5p in the brain between SED‐WT mice and EXE‐WT mice (Figure S7b). To further validate this point, we applied RNA chromogenic in situ hybridization (CISH) assay to visualizing miR‐532‐5p expression in the whole brain. We observed that AD led to a marked reduction in miR‐532‐5p expression (SED‐5XFAD vs SED‐WT). MiR‐532‐5p in the brain presented a 28% higher expression in EXE‐5XFAD mice than in SED‐5XFAD mice, indicating that exercise elevated the expression of miR‐532‐5p in the whole brain of 5XFAD mice (Figure 5d,e). EXE‐WT mice displayed a weakly increased miR‐532‐5p level in the brain compared with SED‐WT mice. This is in agreement with prior observations in RT‐qPCR analysis. Neurons and astrocytes, the most abundant cells in the brain, provided both structural and functional support to the BBB (Xu et al., 2017). Using CISH combined with dual fluorescence staining of TUJ and GFAP, markers of the neuron and astrocyte, we found that, regardless of the cortex or hippocampus, the level of miRNA‐532‐5p in neurons was much higher in EXE‐5XFAD mice than in SED‐5XFAD mice, while there were no apparent changes in miR‐532‐5p in astrocytes after exercise (Figure 5f–h). In addition, we also explored changes in miR‐532‐5p in pericytes and endothelial cells using CISH combined with dual fluorescence staining of PDGFRβ and CD31. The results showed greater miR‐532‐5p around the pericytes and endothelial cells in EXE‐5XFAD mice. The abundance of miR‐532‐5p in pericytes or endothelial cells of EXE‐5XFAD mice was significantly higher than that of SED‐5XFAD mice (both in cortex and hippocampus; Figure S6c–e). However, we did not find an obvious difference in miR‐532‐5p levels in pericytes and endothelial cells.

### 2.6 MiRNA‐532‐5p regulates BBB function via inhibition of *EPHA4*

**FIGURE 6:**
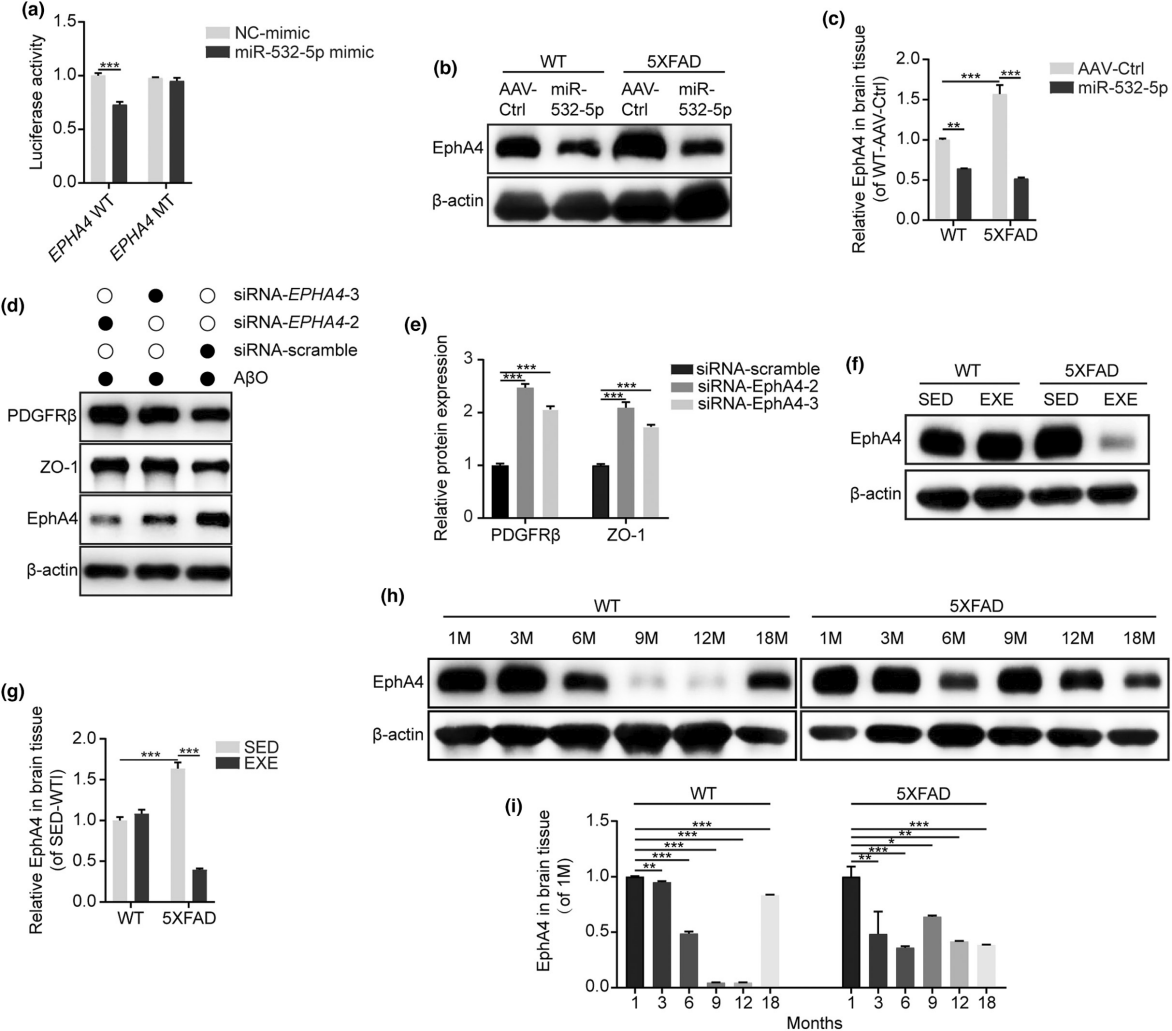
*EPHA4* was a target gene of miR‐532‐5p. (a) Luciferase activity at 48 h post‐co‐transfection of HEK293T cells with miR‐532‐5p mimics and the following plasmids: 3′UTR‐*EPHA4* WT and 3′UTR‐*EPHA4* MT (with mutation in putative miRNA target site). *N* = 3 replicates; Mean ± SEM; ***p < 0.001 by two‐way ANOVA. (b) Representative blots showing the expression of EphA4 in miR‐532‐5p overexpressed mice. (c) Quantification of EphA4 relative expression in miR‐532‐5p overexpressed mice. β‐actin was used as a control. *N* = 4 mice per group; Mean ± SEM; **p < 0.01, ***p < 0.001 by two‐way ANOVA. (d) Representative blots showing protein expressions in primary pericytes and endothelial cells transfected with siRNA of EPHA4 (siRNA‐EPHA4) or siRNA negative control (siRNA‐scramble). (e) Quantification of PDGFRβ and ZO‐1 relative expression by grey values analysis of immunoblots. β‐actin was used as a control. *N* = 3 replicates; Mean ± SEM; ***p < 0.001 by one‐way ANOVA. (f) Representative blots of EphA4 in the brain tissue of 6‐month‐old SED‐WT, EXE‐WT, SED‐5XFAD, and EXE‐5XFAD mice. (g) Quantification of EphA4 relative expression in exercised (EXE‐WT and EXE‐5XFAD) and sedentary (SED‐WT and SSED‐5XFAD) mice. β‐actin was used as a control. *N* = 7 mice per group; Mean ± SEM; ***p < 0.001 by two‐way ANOVA. (h) Representative blots of EphA4 in the brain tissue of WT (left) and 5XFAD (right) mice showing an age‐dependent decline. (i) Quantification of age‐related changes in EphA4 relative expression in WT and 5XFAD mice. β‐actin was used as a control. *N* = 3 mice per group; Mean ± SEM; **p < 0.01, **p < 0.01, ***p < 0.001 by one‐way ANOVA.

A total of 31 target genes of miR‐532‐5p were extracted by the intersection of the gene lists from three different databases, including starbase, miRDB, and TargetScan (Figure S7a). Of them, previous studies demonstrated elevated levels of active Eph receptor A4 (EphA4) in human AD brains. Limitation of downstream effects of EphA4 signaling in neurons could attenuate cognitive impairment associated with Aβ‐induced neurodegeneration in AD (Huang et al., 2017). In addition, activation of EphA4 also contributed to BBB damage or cerebral blood flow through Rho/ROCK or EphA4‐Tie2 signaling, respectively (Chen et al., 2018; Okyere et al., 2020). Thus, we finally focused on *EPHA4* for further analysis. We performed a dual‐luciferase reporter assay to confirm whether miR‐532‐5p was directly bound to the 3′ UTR of *EPHA4*. Compared with the negative control (NC) group, miR‐532‐5p mimics lowered the luciferase activity for putative target genes, whereas luciferase activity for the mutation of a putative miR‐532‐5p binding site within *EPHA4* showed no significant difference (Figure 6a). Moreover, overexpression of miR‐532‐5p in the brain resulted in a reduction in the level of EphA4 in 5XFAD mice and WT mice when compared with control (Figure 6b,c). Having demonstrated that *EPHA4* is under the direct inhibitory control of miR‐532‐5p, we investigated whether *EPHA4* could affect the expression of functional proteins in pericytes and endothelia cells. Three small interfering RNAs (siRNA) were used to silence *EPHA4* expression. siRNA‐2 and siRNA‐3 knocked down EphA4 with the greatest efficiency and were used in subsequent experiments (Figure S7b). Silencing *EPHA4* using siRNA‐2 and siRNA‐3 resulted in an elevated level of PDGFRβ in primary pericytes and ZO‐1 in primary endothelial cells, indicating that *EPHA4* expression could influence the function of these cells (Figure 6d,e). Most remarkably, an increased expression of EphA4 in SED‐5XFAD mice to 63% of that in SED‐WT mice was observed at 6 months of age. After long‐term exercise, we detected a decreased expression of EphA4 in EXE‐5XFAD mice (Figure 6f,g). Furthermore, we observed that EphA4 expression in the brain of WT mice exhibited an age‐dependent reduction during 1–12 months of age but was elevated again in the very old stage (18 months of age). Unlike the changing trend in WT mice, EphA4 in 5XFAD mice increased again after 6 months of age, earlier than that in WT mice (Figure 6h,i).

## 3 DISCUSSION

In this study, we show that long‐term exercise rescues BBB function, including the loosening of tight junctions, the loss of pericytes, and the expression of LRP1 in endothelial cells and pericytes, to lower brain Aβ burden and improve memory abilities in 5XFAD mice. Notably, we identify exercise‐derived brain exosomal miR‐532‐5p as a mediator to convey protective signals of exercise to the BBB system.

Impairment of BBB structure and function is a preclinical feature of AD pathology (Cai et al., 2018). Indeed, in our study, multiple changes to the BBB in SED‐FAD mice were observed, including loosed tight junctions, impaired pericyte coverage, and reduced expression of LRP1 in BBB components. After long‐term exercise, the function of the BBB in EXE‐5XFAD mice was significantly improved (Soto et al., 2015). Consistent with these results, we also observed a significant increase in Aβ efflux across the BBB, suggesting that improved BBB function resulted from long‐term exercise could increase the clearance of Aβ (Storck et al., 2016). Cerebral blood flow (CBF) reductions have also been a hallmark in patients with AD (Duan et al., 2020). However, our studies did not show any difference in CBF after exercise (van der Kleij et al., 2018). This is more likely due to the fact that our experiment only reveals the CBF in the macrovascular and not in the microvascular system.

Exercise enhances the exosomes biogenesis, making changes in amounts and contents (Fruhbeis et al., 2015; Nair et al., 2020), which benefits various diseases (Wang et al., 2020). In our study, we found that the exosomes isolated from brain tissue could be internalized by primary pericytes and endothelial cells to exert profound cell protective effects against AβO injury, as evidenced by increased expression of functional proteins such as PDGFRβ and ZO‐1, reduced apoptotic and improved cell survival. These results suggest that exosome cargoes seem to be active and may act as a mechanism to spread exercise‐induced changes across the BBB.

We screened the alterations in five candidate miRNAs associated with vascular dysfunction (Harati et al., 2022; Kazenwadel et al., 2010; Slater et al., 2018; Tang et al., 2017; Xu et al., 2017). Among those miRNAs, miR‐532‐5p was of particular interest because it showed the most dramatic change in the brain exosomes of EXE‐FAD mice, compared with SED‐FAD mice. The involvement of miR‐532‐5p in various pathological processes has been well documented (Bayoumi et al., 2017; Wang et al., 2020). In our study, we observed that miR‐532‐5p was downregulated in both brain tissue and brain exosomes of 5XFAD mice, consistent with the changes in miR‐532‐5p in AD patients (Ludwig et al., 2019) and in serum exosomes of AD mice (Su et al., 2022). After long‐term exercise, we found that the level of miR‐532‐5p in brain exosomes was significantly increased. More importantly, the elevated miR‐532‐5p increased PDGFRβ, ZO1, NG2, and LRP1 expression both in vitro and in vivo, which in turn facilitate Aβ clearance (Slater et al., 2018). In addition, an age‐dependent loss of PDGFRβ and ZO1 in 5XFAD mice was observed starting from 3 to 6 and 1–3 months of age, respectively, earlier than in WT mice (6–9 months of age; Giannoni et al., 2016). Meanwhile, miR‐532‐5p in 5XFAD mice declined from 1–3 months of age, also earlier than in WT mice (12–18 months of age). Thus, the onset of BBB compromise was earlier in 5XFAD mice than in normal aging. Consistent with these results, we observed significant correlations between age‐dependent loss of PDGFRβ and ZO1 and age‐related changes in miR‐532‐5p from 1 to 18 months of age in 5XFAD. While for WT mice, we speculate that the remarkable effect of miR‐532‐5p on BBB dysfunction in normal aging may be observed after 6–9 months of age. We also investigated the origins of the exercise‐induced increase in exosomal miR‐532‐5p. It was found that long‐term exercise significantly increased miR‐532‐5p in brain tissue but not in the muscle, liver, or kidney. Notably, neurons were partly responsible for the significantly higher level of miR‐532‐5p in the brain, suggesting that neural activity may communicate with brain vascular function (Xu et al., 2017).

Based on bioinformatics and luciferase reporter assay, we identified *EPHA4* as a target of miR‐532‐5p. We detected brain EphA4 downregulation in treadmill‐trained and miR‐532‐5p overexpressed 5XFAD mice, indicating that this reduction depends on the changes of miRNAs induced by exercise. In vitro experiments further highlighted the remedial effects of knockdown EphA4 on the function of BBB‐associated cells under an AD environment (Fan et al., 2017; Okyere et al., 2020). All these results support that an increased level of miR‐532‐5p induced by exercise results in the downregulation of EphA4 expression, which in turn may rescue the function of the BBB in 5XFAD mice. However, we did not find a correlation between miR‐532‐5p and EphA4 as a function of age. EphA4 is the key component in axon and vascular guidance in the developing CNS (Goldshmit et al., 2006). Thus, at the earlier age (1–3 months of age), a higher expression of EphA4 was observed in the brain of both WT and 5XFAD mice, although the expression of miR‐532‐5p begun to decline (Figure 6d,e). From 6 months of age, the brain tissues that showed higher expression of EphA4 also showed a lower level of miR‐532‐5p. For example, in WT mice, we observed higher levels of miR‐532‐5p at 6–12 months of age, which declined at 18 months of age. Correspondingly, lower expression of EphA4 at 6–12 months of age but higher at 18 months of age was found (Figure S5h and Figure 6d). In 5XFAD mice, the level of miR‐532‐5p was slightly increased at 9 months of age but lower at 12 or 18 months of age than that at 6 months of age. Consistently, the expression of EphA4 was higher at 12 or 18 months of age than that at 6 months of age (Figure S5e and Figure 6d). The increased expression of EphA4 at 9 months of age could be partially due to the compensatory effect.

Our study has some limitations. First, although our results suggest that neurons are part of the source of exercise‐induced exosomal miR‐532‐5p, complete identification of the origins of exosomal miR‐532‐5p awaits further elucidation due to the inadequate understanding of the exosomal RNA sorting process and the complexity of brain cell‐to‐cell communication. Second, the fact that miR‐532‐5p can decrease EphA4 expression suggests that the miRNA exerts at least part of its effect through EphA4. However, because miRNAs regulate a wide spectrum of genes, we cannot rule out the possibility of other miR‐532‐5p targets.

Overall, our study shows that long‐term exercise triggers the release of exosomes by the brain, carrying an increased level of miR‐532‐5p that induces gene expression changes in the BBB, leading to an improved BBB function in AD. These results suggest that improving the function of the BBB through exosomal miR‐532‐5p may be a potential therapeutic strategy for AD.

## 4 METHODS

### 4.1 Animals and treadmill protocols

Transgenic male mice carrying five mutations (APPSwF1Lon, PSEN1*M146L*L286V) associated with early onset familial Alzheimer's disease (5XFAD mice; Oakley et al., 2006) and C57BL/6J wild‐type male mice (WT mice) were used in all experiments. Mice were divided into four groups: SED‐WT, EXE‐WT, SED‐5XFAD, and EXE‐5XFAD. The treadmill test was performed starting with 2‐month‐old mice, five times a week for 16 consecutive weeks (Cho et al., 2015). For the first 5 days, the velocity is 2 m/min for 5 min, 5 m/min for 5 min, and 8 m/min for 20 min. For the subsequent weeks, the velocity is 8 m/min for 5 min, 14 m/min for 20 min, and 16 m/min for 5 min. Mice in the sedentary groups were left on the treadmill without running for the same duration of time. All animal experiments were performed in strict accordance with the protocols approved by the Institutional Animal Care and Research Advisory Committee of Shandong University, Jinan, Shandong, China.

### 4.2 MWM test

Spatial learning and memory were evaluated by the MWM, following a protocol, adapted from previous reports (Belaya et al., 2020). The escape latencies, swimming speed, and platform‐crossing times were recorded using an automated video tracking system (Smart 3.0 Premium, Harvard/Panlab, USA).

### 4.3 Eight‐arm radial maze

The whole experiment included three sessions, operated as previously reported with some modifications (Prades et al., 2017). The frequency of working memory and reference memory errors were evaluated.

### 4.4 Immunohistochemical and immunofluorescence

Both paraffin and frozen sections were employed for the experiment. After being dried, dewaxed, hydrated, and undergoing high‐pressure antigen repair, paraffin‐embedded brain sections (4 μm) were incubated with peroxidase blocker for 10 min at room temperature. The sections were washed with PBS and were then incubated overnight with anti‐4G8 (1:800 dilution, 800712, BioLegend, San Diego, CA, USA) at 4°C. Sections were washed and incubated with hypersensitive enzyme‐labeled goat anti‐mouse polymer. Positive reactions were visualized with a 3,3N‐Diaminobenzidine tetrahydrochloride (DAB)‐peroxidase substrate and counterstained with hematoxylin for 1 min. Finally, stained sections were dehydrated through graded alcohols, cleared in xylene, and sealed with neutral resin.

For immunofluorescence, frozen sections (12 μm) were washed with PBS, permeabilized with 0.3% Triton‐X‐100, blocked with 1% bovine serum albumin for 30 min, and then incubated with the following primary antibodies overnight at 4°C: anti‐PDGFRβ antibody (1:200 dilution, ab32570, Abcam, Cambridge, UK), anti‐ZO1 antibody (1:50 dilution, 61‐7300, Thermo Fisher Scientific, MA, USA), anti‐claudin5 antibody (1:50 dilution, #35‐2500, Thermo Fisher Scientific, MA, USA), anti‐LRP1 antibody (1:100, sc57353, Santa Cruz Biotechnology, TX, USA), and anti‐CD31 antibody (1:100 dilution, AF3628, R&D systems, MN, USA). PBS in the absence of primary antibody was used as a negative control. On the next day, sections were washed and incubated with an appropriated secondary antibody for 1 h at 37°C. Finally, the sections were incubated with 4′,6‐diamidino‐2‐phenylindole (DAPI) for 5 min, washed in PBS, and sealed with the anti‐fluorescence quencher.

### 4.5 Western blot analysis

Protein samples were separated by SDS‐PAGE gel electrophoresis and transferred to a PVDF membrane. Membranes were blocked in 5% milk, and incubated with the following primary antibodies overnight at 4°C: anti‐PDGFRβ antibody (1:1000 dilution, ab32570, Abcam, Cambridge, UK), anti‐ZO1 antibody (1:1000 dilution, 61‐7300, Thermo Fisher Scientific, MA, USA), anti‐claudin‐5 antibody (1:1000 dilution, #35‐2500, Thermo Fisher Scientific, MA, USA), anti‐LRP1 antibody (1:50,000, ab92544, Abcam, Cambridge, UK), anti‐NG2 antibody (1:1000, ab129051, Abcam, Cambridge, UK), anti‐EphA4 antibody (1:500 dilution, #37‐1600, Thermo Fisher Scientific, MA, USA). After primary antibody incubation, the appropriate horseradish enzyme labeling antibody (Zhongshan Golden Bridge Biotechnology Co., Ltd., Beijing, China) was used as a secondary antibody. Proteins were visualized using an ECL reagent (Millipore).

### 4.6 Measurement of BBB permeability

Blood–brain barrier permeability was assessed using the EB assay. Mice were administered EB (0.5%, DK0001, Leagene Co., Ltd, Beijing, China) through tail vein injection and allowed to circulate for 2 h. Subsequently, mice were perfused with PBS to completely remove the EB in blood vessels. The brains were carefully dissected and weighted. Next, the brains were homogenized using formamide at a ratio of 1 g tissue to 2 ml formamide. The homogenates were incubated in a 50°C water bath for 72 h to extract the Evans Blue dye. The absorbance of the extract was measured with a spectrophotometer at 620 nm. The dye concentration in extracts was calculated using a standard curve of Evans Blue in formamide and normalized to the brain weight. EB extravasation was also detected as red fluorescence in frozen coronal brain sections after administration as described previously.

### 4.7 Purification, identification, and labeling of exosomes

Purification of exosomes was achieved by differential centrifugation and sucrose density gradient centrifugation as previously described with some modifications (Perez‐Gonzalez et al., 2017). Finally, exosome pellets were resuspended in PBS for further analysis, including NTA, electron microscopy examination, Western blot analysis, and PKH26 red fluorescent labeling.

### 4.8 miRNA preparation and quantitative reverse transcription PCR (RT‐qPCR)

Total miRNAs of murine brains, isolated exosomes and cultured cells were extracted by a miRcute miRNA Isolation Kit (#DP501, TIANGEN BIOTECH Co., Ltd., Beijing, China) according to the manufacturer's instructions. The extracted miRNAs were reversely transcribed into cDNAs using a poly‐A tailing method conducted by the Mir‐X™ miRNA First‐strand synthesis (638313, Takara). The real‐time PCR assays were performed using the SYBR Green PCR Kit. The relative miRNA amount was determined by the 2^−ΔΔCt^ method and normalized with U6 expression (as an internal control). The primers used for real‐time PCR are as follows.

mmu‐miR‐27b‐3p primer: 5′‐UUCACAGUGGCUAAGUUCUGC‐3′
mmu‐miR‐532‐5p primer: 5′‐CAUGCCUUGAGUGUAGGACCGU‐3′
mmu‐miR‐181a‐5p primer: 5′‐AACAUUCAACGCUGUCGGUGAGU‐3′
mmu‐miR‐132‐3p primer: 5′‐UAACAGUCUACAGCCAUGGUCG‐3′
mmu‐miR‐124‐3p primer: 5′‐UAAGGCACGCGGUGAAUGCC‐3′

### 4.9 Surgical procedures

Animals were anesthetized and then fixed in a stereotaxic frame. The hair on the head was shaved, and the skin was cleaned before making a small incision to expose the skull. Mice received bilateral injections of virus solution (GPAAV‐CMV‐mmu‐miR‐532‐5p‐PGK‐T2A‐eGFP‐WPRE) into the lateral ventricle using coordinates of −0.8 mm posterior, ±1.0 mm lateral, and − 2.4 mm ventral from Bregma. The virus solutions with a total volume of 5 μl were injected using a 10 μl syringe over 10 min and the syringe was left in place for an additional 10 min to allow diffusion of the virus solution. The needle was then slowly removed from the brain and the incision was sutured.

### 4.10 Aβ clearance via the BBB

Brain clearance of human Aβ42 peptides was determined simultaneously with inulin (reference marker) in SED‐WT mice and EXE‐WT mice using a procedure as described (Zhao et al., 2015). Briefly, 0.5 μl of test molecules (1.4 ng/μl of Aβ42, 22.2 mg/ml of inulin) was administered to the right caudate nucleus (0.9 mm anterior, 1.9 mm lateral and 2.9 mm below the surface of the bregma). Brain and blood were sampled 30 min after the Aβ42 injection and prepared for Aβ42 ELISA (Invitrogen, KHB3441) according to the kit instructions. Samples were diluted 1:1 before added to ELISA plates. The retention of inulin was determined by UV–Visible spectroscopy. The percentage of Aβ42 or inulin in the brain was calculated as 100× (*N*_*b*_/*N*_*i*_), where *N*_*b*_ is the amount of Aβ42 or inulin remaining in the brain at the end of the experiment and *N*_*i*_ is the amount of Aβ42 or inulin injected into the brain. The percentage of Aβ42 cleared through the BBB was calculated as [(1 − *N*_*b*_(Aβ42)/*N*_*i*_(Aβ42)) – (1 − *N*_*b*_(inulin)/*N*_*i*_(inulin))] × 100.

### 4.11 Cell transfections

Primary pericytes and endothelial cells were isolated from 8‐week‐old C57BL/6J mice, according to the protocol of relevant research (Bernard‐Patrzynski et al., 2019). Afterward, primary cells were maintained in the iCell Primary Cell Culture System. Briefly, cells were pretreated with Aβ oligomer (AβO) (3 μM) to simulate AD‐associated environment and then transfected using lipofectamine iMAX transfection reagent (Invitrogen) with miRNA‐532‐5p mimics (10 μM) or inhibitors (20 μM) to overexpression or decrease the level of miRNA‐532‐5p, interfering RNAs (siRNAs) (control and *EPHA4*) (Genomeditech Co., Ltd., Shanghai, China) to silence the expression of the *EPHA4* gene.

### 4.12 PI staining

Cell apoptosis was assessed by PI staining. The cells were washed twice with cold PBS, and then stained with PI (10 μM) for an additional 20 min. DAPI was used to stain cell nuclei for 5 min.

### 4.13 MTT assay

Cells were incubated for 4 h at 37°C with 1 mg/ml of MTT, dissolved in DMEM, rinsed with PBS, and then treated with DMSO (200 μl). The absorbance was recorded at 540 nm using a microplate reader.

### 4.14 MiR‐532‐5p in situ hybridization

The localization of miR‐532‐5p in brain sections was investigated using the miR NAscope HD Detection Kit (Advanced Cell Diagnostics, Hayward, CA, USA), according to the manufacturer's protocol. In brief, 10‐μm‐thick frozen sections were pretreated and hybridized with ZZ‐probe targeting miR‐532‐5p for 4 h. Six amplification steps were used to detect ZZ‐pairs binding miR‐532‐5p before visualizing with Fast Red solution.

### 4.15 Luciferase reporter assay

The sequence of EPHA4 was searched from GenBank, and the 3′UTR of EPHA4 was cloned into a specific vector (Genomeditech Co., Ltd., Shanghai, China). Either miR‐532‐5p mimics or NC was co‐transfected with the luciferase reporter plasmid PGL3‐CMV‐LUC‐Mouse_EPHA4 3′UTR vector. At 48 h after transfection, luciferase activity was measured by the Dual‐Luciferase reporter assay system. The relative luminescence (Firefly/Renilla) was calculated across three replicates. For each replicate, values were further normalized to those of the control setup.

### 4.16 Statistical analyses

Data were analyzed using the GraphPad Prism 8.0 software (GraphPad Software Inc., San Diego, CA, USA) and IBM SPSS Statistics version 26.0 (IBM Corp., Armonk, NY, USA). Differences between the two groups were determined by a two‐tailed Student's *t*‐test. We used one‐way ANOVA and two‐way ANOVA for multiple‐group comparisons. Correlation analyses were performed by Pearson regression. All *n* and *p* values, as well as statistical tests, are displayed in the figure legends.

## AUTHOR CONTRIBUTIONS

Y.D., T.H., and X.L. conceived the study. X.L. wrote the manuscript. T.H., W.F., and C.Q. contributed to writing the manuscript. T.H. and X.L. planned and carried the experiments. W.F., N.W., Y.P., C.L., M.Z., N.T., X.H. Y.W. contributed to the experiments. All authors have contributed to, read, and approved the final manuscript.

## CONFLICT OF INTEREST

The authors declare no financial or other conflicts of interest.

## Supporting information


FigureS1

FigureS2

FigureS3

FigureS4

FigureS5

FigureS6

FigureS7

supinfo

## Data Availability

The data that support the findings of this study are available from the corresponding author upon reasonable request.
