# Supplementary figures and images for: Exosomal miR‐532‐5p induced by long‐term exercise rescues blood–brain barrier function in 5XFAD mice via downregulation of EPHA4
Source: Aging Cell. 2022 Dec 9;22(1):e13748. doi: 10.1111/acel.13748 (PMC9835579; doi:10.1111/acel.13748)

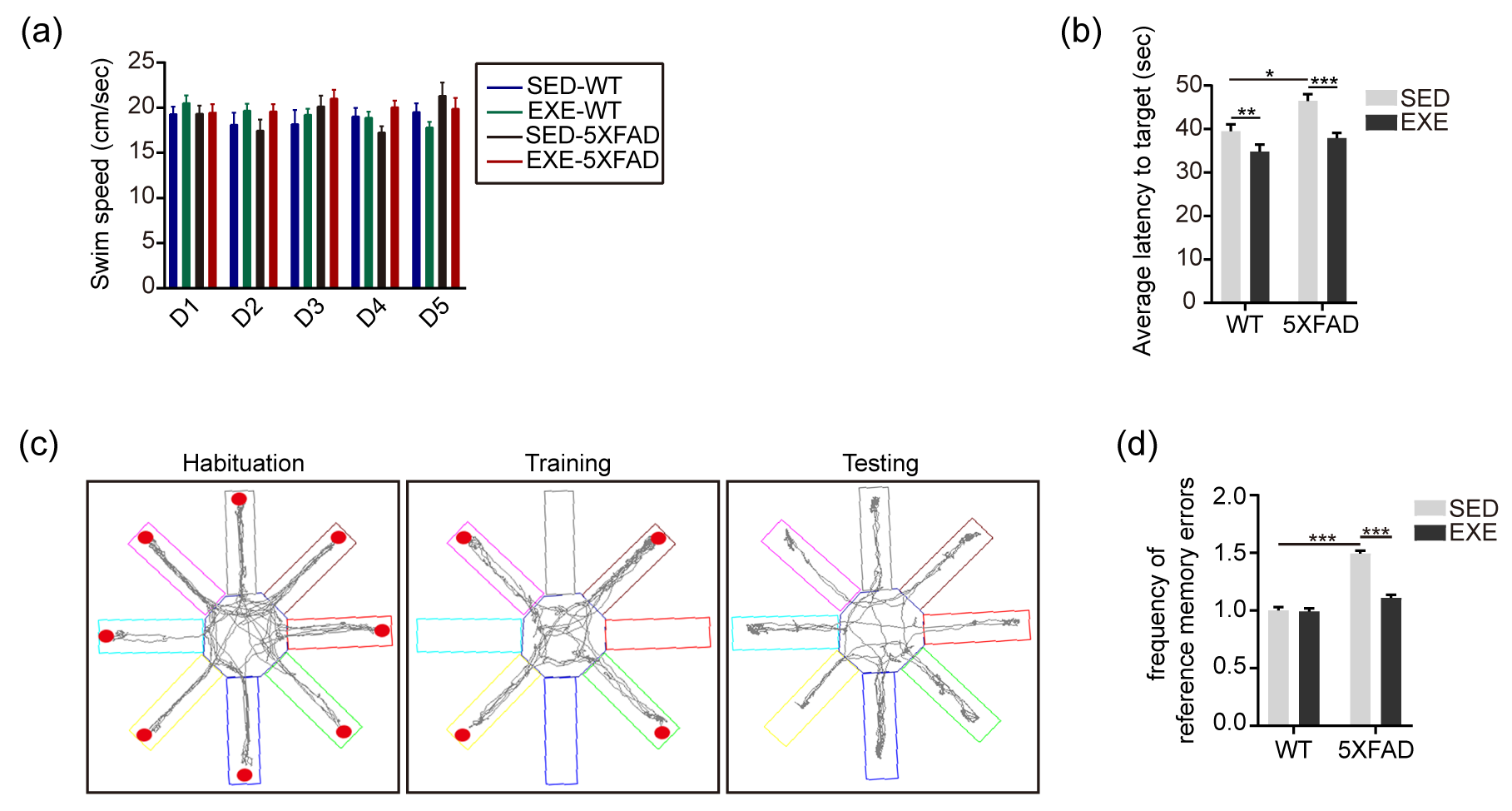

Supplement: Supplementary file 1 — FigureS1 [file ACEL-22-e13748-s008.tif]

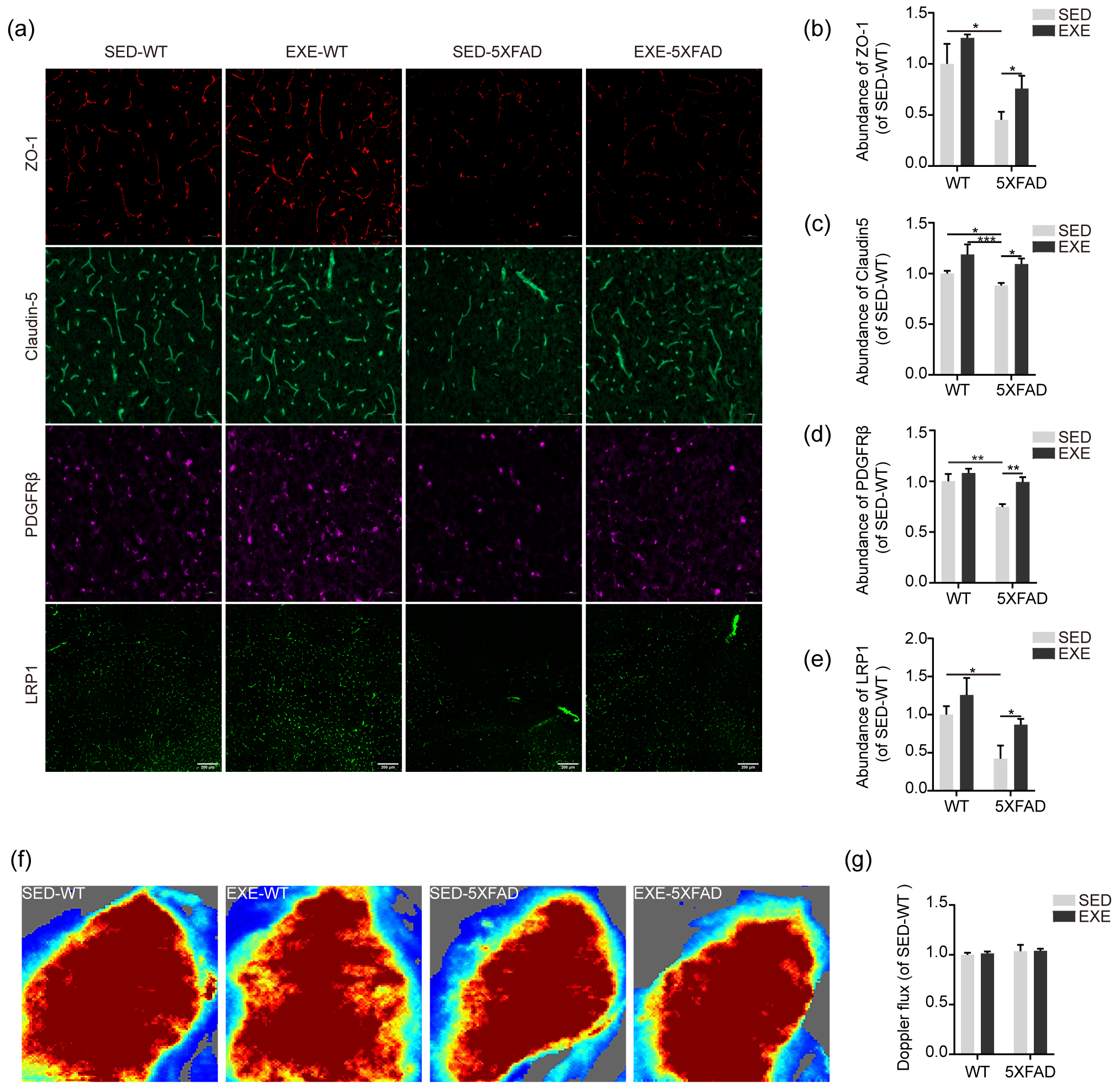

Supplement: Supplementary file 2 — FigureS2 [file ACEL-22-e13748-s007.tif]

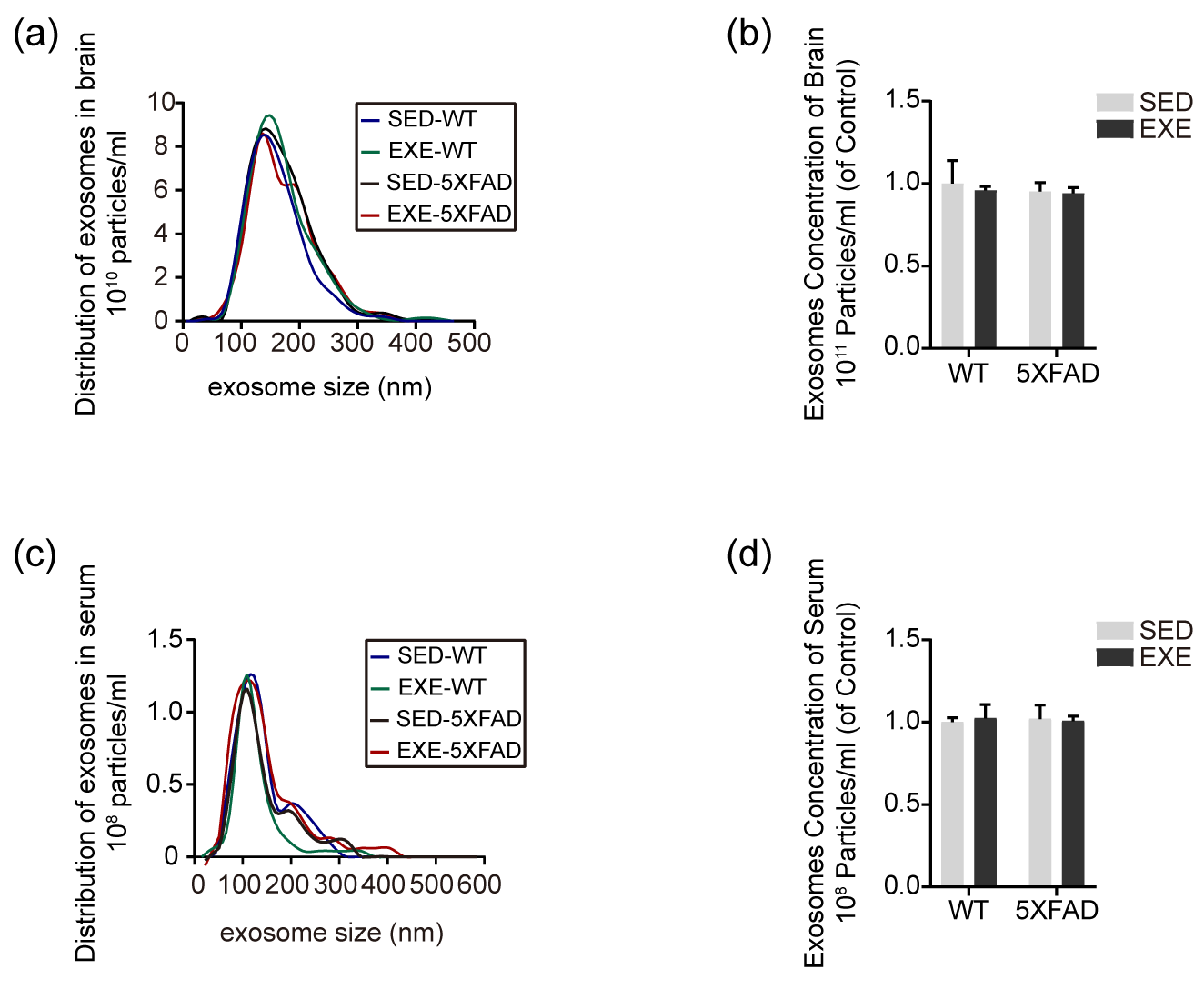

Supplement: Supplementary file 3 — FigureS3 [file ACEL-22-e13748-s003.tif]

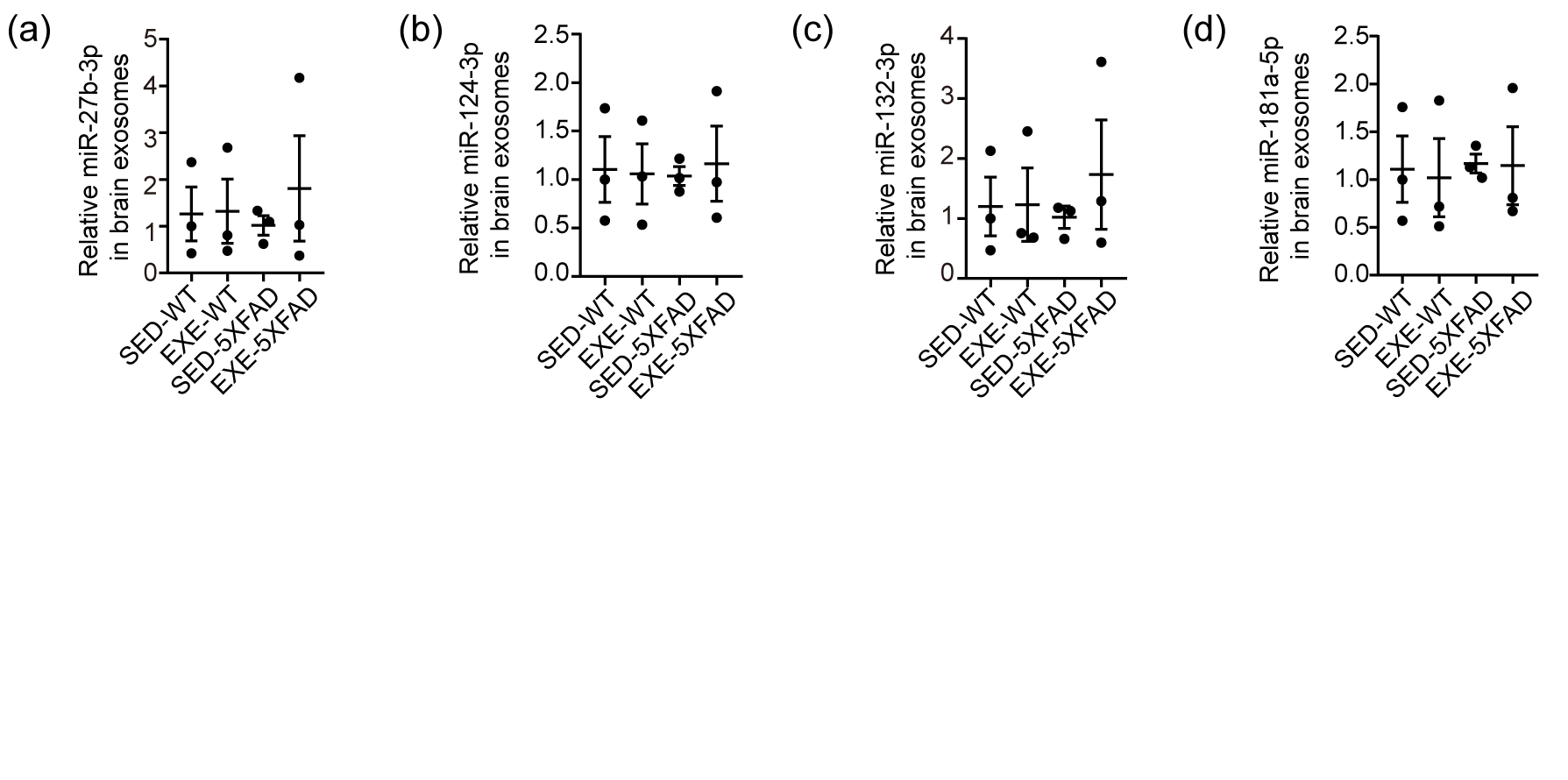

Supplement: Supplementary file 4 — FigureS4 [file ACEL-22-e13748-s006.tif]

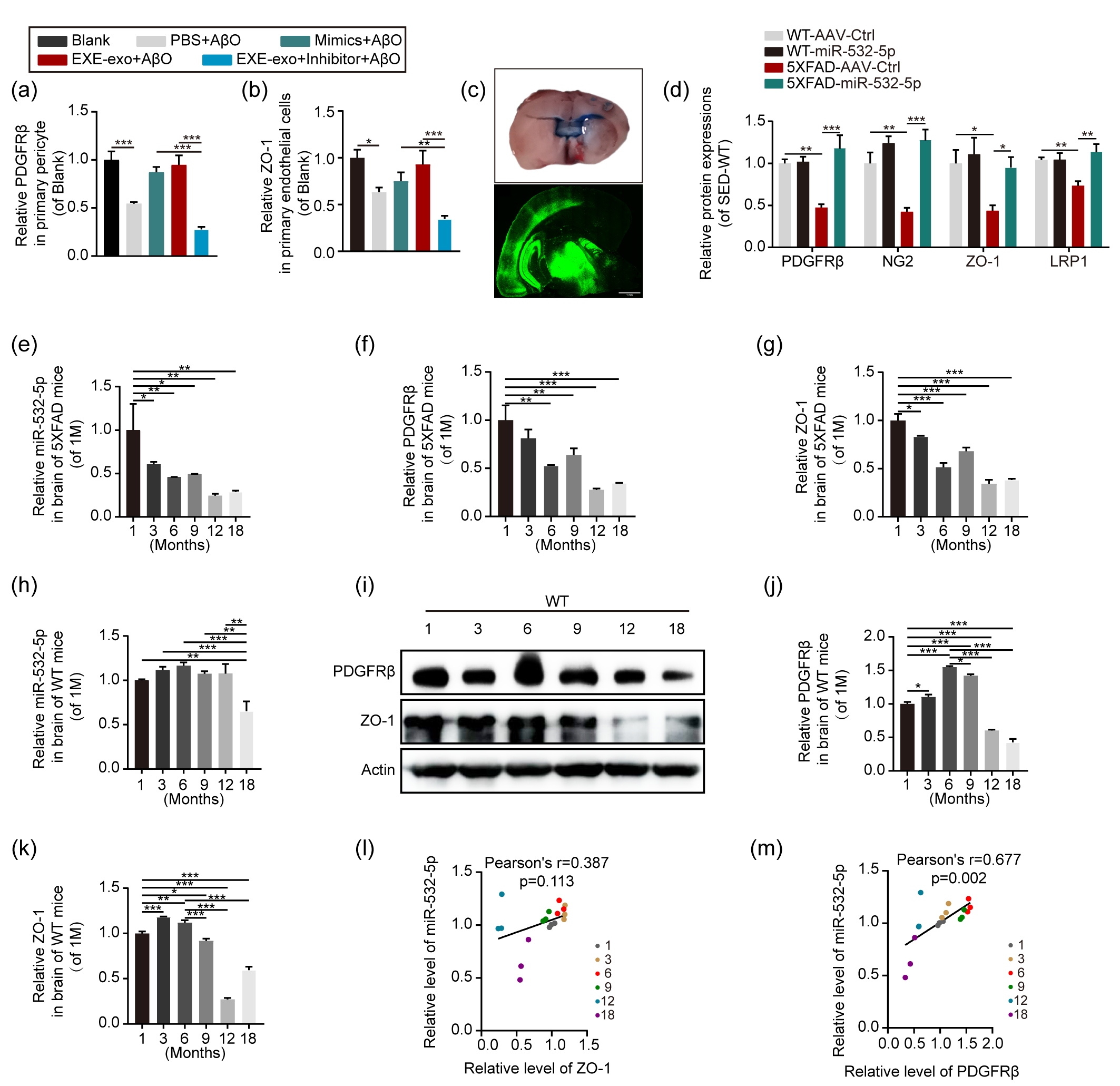

Supplement: Supplementary file 5 — FigureS5 [file ACEL-22-e13748-s002.tif]

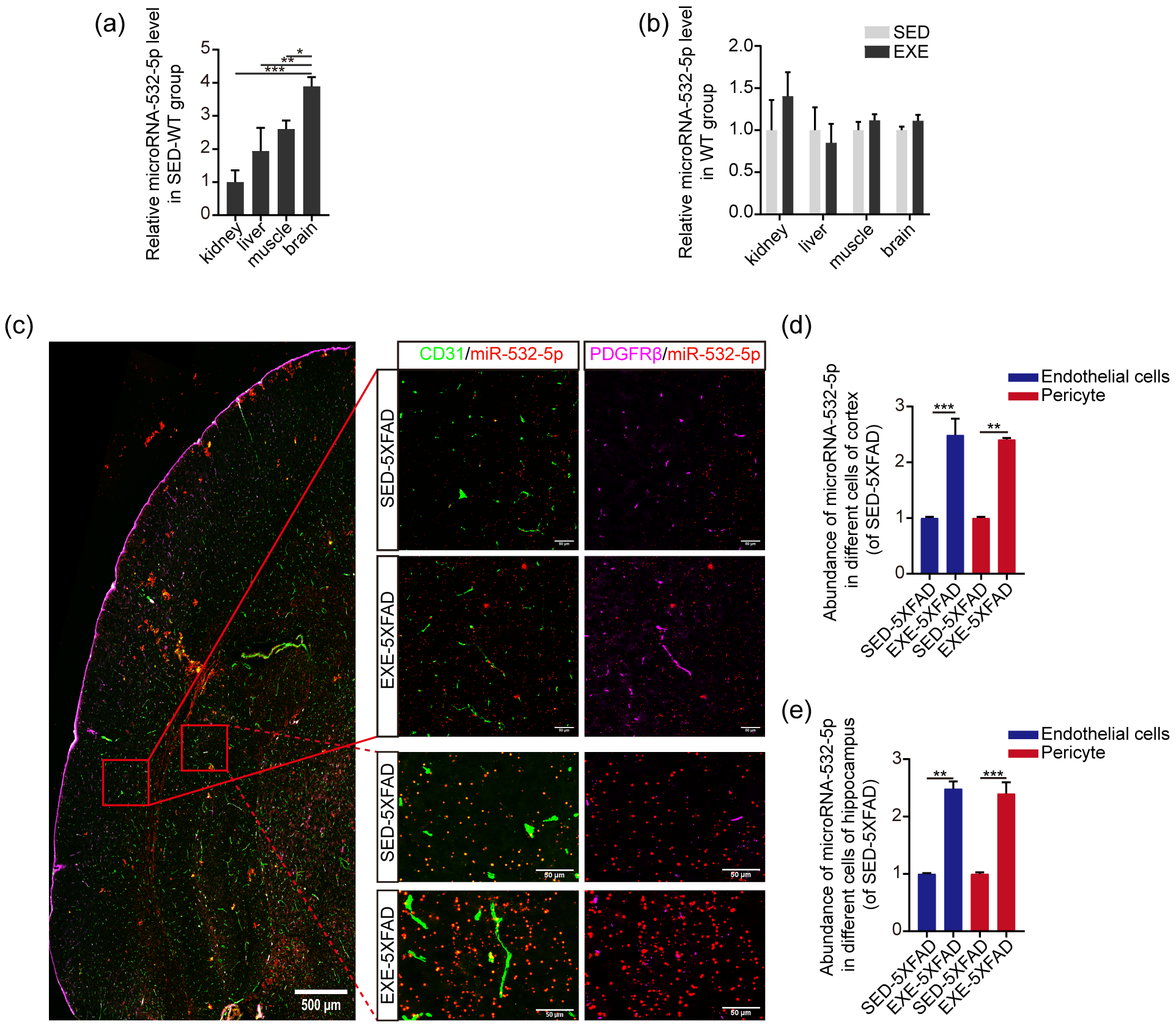

Supplement: Supplementary file 6 — FigureS6 [file ACEL-22-e13748-s001.tif]

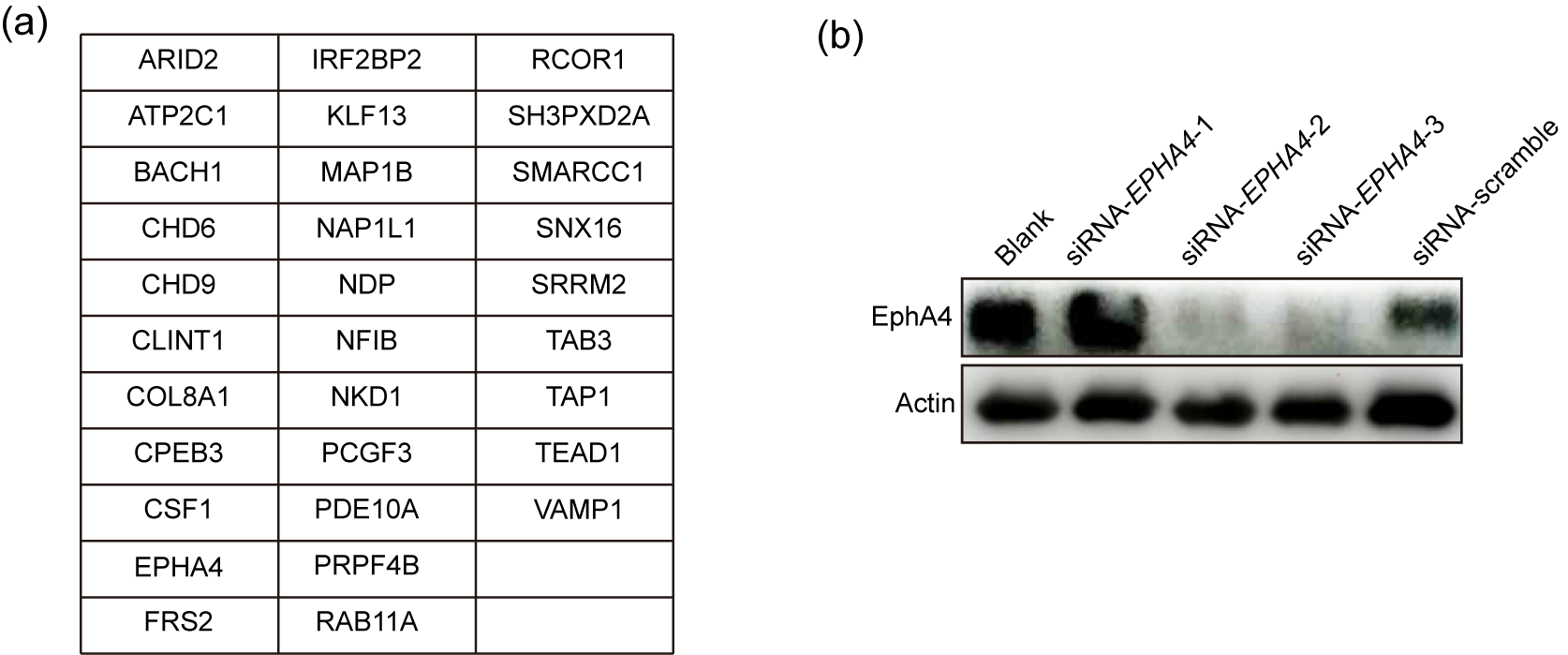

Supplement: Supplementary file 7 — FigureS7 [file ACEL-22-e13748-s004.tif]
