# Supplementary material for: Exosomal miR‐532‐5p induced by long‐term exercise rescues blood–brain barrier function in 5XFAD mice via downregulation of EPHA4
Source: Aging Cell. 2022 Dec 9;22(1):e13748. doi: 10.1111/acel.13748 (PMC9835579; doi:10.1111/acel.13748)
Supplement: Supplementary file 8 — supinfo [file ACEL-22-e13748-s005.docx]

**Supplementary Methods**

**Eight-arm radial maze**

The radial eight-arm maze apparatus comprised of eight side arms (30cm long and 8cm wide) and an octagonal central area. Animals were put on food restriction (>85% of initial weight) for a week before the beginning of the experiment. The whole experiment included three sessions, operated as previously reported with some modification(Prades et al., 2017). For the two-day habituation session, animals were allowed to explore the wide-open maze with the food pellets (14mg) spread around all the end of arms (90sx3 trials per day). For the training session, initially, animals stayed in the central area with all arms blocked. Food pellets were placed in the four arms after which these four arms were opened. After three training days, all arms were opened. Each animal was trained for 90 seconds three times per day. The trial ended when animals in SED-WT group made over 50% correct choices on two consecutive days. For the test session, removed all food pellets and opened all arms. Frequency of working memory and reference memory errors were assessed.

**Purification, identification and labeling of exosomes**

Briefly, murine hemibrains were finely minced and digested in Hibernate-A (A1247501, Thermo Fisher Scientific Inc., Waltham, MA, USA) containing 1mg/mL of collagenase D (11088858001, Roche Diagnostic, Indianapolis, IN, USA) in a shaking water bath at 37℃ for 20 min, and then stopped the enzymatic reaction by adding protease inhibitors (PMSF 1:100; easy 1:25). All homogenate were centrifuged at 4℃, sequentially at 300g for 10 min to discard brain cells, at 2000g for 10 min to discard large cell debris, at 10000g for 30 min to discard small cell debris. The supernatants were collected carefully, filtered using a 0.22 polyvinylidene fluoride membrane filter and finally ultracentrifuged at 100000g at 4℃ for 70 min for twice (Type 90Ti rotor, Beckman Coulter, Brea, CA, USA). Exosomes pellets were resuspended in 2 mL of 0.95M sucrose solution and then inserted inside a sucrose step gradient column (six steps starting from 2.0M sucrose up to 0.25M sucrose in 0.35M increments). The sucrose step gradient column was ultracentrifuged at 200000g for 16 hours at 4℃ (Type SW41Ti). Seven fractions (a-g) were collected respectively, diluted in cold PBS, and ultracentrifuged at 10000g for 70 min at 4℃. Exosomes pellets were resuspended in PBS, fixed to the copper mesh, stained with Uranyl oxalate and finally examined by electron microscopy.

For up-take studies, purified exosomes were labeled using a PKH26 red fluorescent labeling kit (Sigma-Aldrich, Lot # MKCK8658). Briefly, the resuspended exosomes were added to 150 μl Diluent C, while in parallel 1 μl PKH26 dye was added in 250 μl Diluent C and incubated with the exosome solution for 4 min at room temperature. Then, 500 μl 1% bovine serum was added to bind excess dye. Finally, labeled exosomes were collected by centrifuging at 100000 g for 1 h.

**Nanoparticle tracking analysis (NTA)**

The size and concentration of exosomes were measured by ZetaView system (Particle Metrix GmbH, Inning am Ammersee, Germany) equipped with a 488nm laser and a high-sensitivity complementary metal–oxide–semiconductor (CMOS) camera. Briefly, nanoparticles were captured from scanning 11 cell positions. Data was analyzed by the in-build ZetaView Software 8.05.05 SP2 with specific analysis parameters: Maximum particle size: 1,000, Minimum particle size 5, Minimum particle brightness:10. Triplicate measurements were recorded for each sample.

**Primary pericytes and endotheliocytes isolation and enrichment**

Primary pericytes and endotheliocytes were isolated from 8-week-old C57BL/6J wild-type mice, according to the protocol of relevant research(Bernard-Patrzynski et al., 2019). Briefly, after removing of cerebellum, pons, medulla, meninges and choroid plexuses, the rest brain tissues were homogenized, centrifuged (800g, 8min, 4℃), and digested in DMEM containing typeⅡcollagenase and typeⅠDNase for 75 min at 37℃.Ice-cold DMEM was used to stop the reaction. Afterwards, the homogenate was mixed with DMEM containing 20% BSA and centrifugated at 1000g for 20 min to remove the myelin. The pellet was resuspended in 1 ml of ice-cold DMEM and overlay on the top of cold 33% continuous isotonic Percoll gradient and then centrifuged at 1000g for 10 min. Finally, the micro-vessel layer was collected. To enrich primary endotheliocytes, brain micro-vessels were seeded on 6-well plates previously coated with type Ⅳ collagen and cultured in high glucose DMEM. Primary pericytes were cultured in low glucose DMEM to avoid the proliferation of endotheliocytes.

**Aβ** **oligomers preparation**

AβO was synthesized according to the following steps. First, 1mg lyophilized synthetic human Aβ_1-42_ peptide (ChinaPeptides Co., Ltd; #04010011) was monomerized by dissolving in hexafluoroisopropanol (HFIP) and separated into 10 aliquots in low-binding tubes. Then, HIFP was evaporated at room temperature. 0.1 mg peptide was resuspended in 20 μl DMSO and added to 202 μl medium with a final concentration of 100μM. The peptide solution (100 μM) was then incubated at 4℃ for 24 hours to prepare AβO.

**Cell transfections**

The sequences of miRNA-532-5p inhibitor and miRNA-532-5p mimics were as follows:

miRNA-532-5p inhibitor: 5’-ACGGUCCUACACUCAAGGCAUG-3’

miRNA-532-5p mimics: 5’-CAUGCCUUGAGUGUAGGACCGUGGUCCUACACUCAAGGCAUGUU-3’

The sequences of three siRNA oligo nucleotides were as follows:

Epha4(m)-si-1: 5’-GCAGCACCAUCAUCCAUUGTTCAAUGGAUGAUGGUGCUGCTT-3’

Epha4(m)-si-2: 5’-GCAAUUGCGUAUCGUAAAUUUTTAAAUUUACGAUACGCAAUUGCTT-3’

Epha4(m)-si-3: 5’-CCCGCGAAUGAAGUUACUUUATTUAAAGUAACUUCAUUCGCGGGTT-3’

**Image analysis**

The coronal sections were derived from six-month-old mice. Three slices per mouse were used for Aꞵ staining. Image of Aꞵ staining was visualized at 20X magnification using a Zeiss Axio Imager Z2 Microscope System (Carl Zeiss, Jena, Germany) and acquired using a TissueFAXS plus (TissueGnostics GmbH, Vienna, Austria). Aꞵ plaque was defined as a cluster of granules which was 4G8^+^. The whole right hemisphere of coronal sections was selected and measured using Image J.

**References**

Bernard-Patrzynski, F., Lecuyer, M. A., Puscas, I., Boukhatem, I., Charabati, M., Bourbonniere, L., Ramassamy, C., Leclair, G., Prat, A. & Roullin, V. G. 2019. Isolation of endothelial cells, pericytes and astrocytes from mouse brain. *PLoS One,* 14**,** e0226302.

Prades, R., Munarriz-Cuezva, E., Uriguen, L., Gil-Pisa, I., Gomez, L., Mendieta, L., Royo, S., Giralt, E., Tarrago, T. & Meana, J. J. 2017. The prolyl oligopeptidase inhibitor IPR19 ameliorates cognitive deficits in mouse models of schizophrenia. *Eur Neuropsychopharmacol,* 27**,** 180-191.

**Figure legends**

**Figure S1. The differences in swimming speed, average latency to target and the frequency of reference memory errors between groups. Related to Figure 1**

(a) Comparison of swimming speed between four groups. N=19 mice per group.

(b) Average latency to target showing the average time to reach the platform in five days. N=19 mice per group; Mean ± SEM; **p<0.01 by two-way ANOVA.

(c) Illustration of the Eight-arm radial maze paradigm. In the habituation trials, all arms were open and baited with rewards. In the training trials, four arms were blocked. In the testing trials, all arms were open without rewards.

(d) Comparison of the frequency of reference memory errors between four groups. N=8 mice per group; Mean ± SEM; ***p<0.001 by two-way ANOVA.

**Figure S2. Long-term exercise improves BBB integrity in 5XFAD mice. Related to Figure 2**

(a) Representative confocal microscopy images for ZO-1 (red), Claudin-5 (green), PDGFRβ (magenta), and LRP1 (green) in the whole brain of 6-month-old SED-WT, EXE-WT, SED-5XFAD and EXE-5XFAD mice. Scan bar: 50μm for ZO-1 and Claudin-5, 20μm for PDGFRβ, 200μm for LRP1.

(b) Quantification of abundance of ZO-1 by fluorescence intensity analysis in WT and 5XFAD mice. N=5 mice per group; Mean ± SEM; *p<0.05 by two-way ANOVA.

(c) Quantification of abundance of Claudin-5 by fluorescence intensity analysis in WT and 5XFAD mice. N=5 mice per group; Mean ± SEM; *p<0.05, ***p<0.001 by two-way ANOVA.

(d) Quantification of abundance of PDGFRβ on pericytes by fluorescence intensity analysis in WT and 5XFAD mice. N=5 mice per group; Mean ± SEM; **p<0.01 by two-way ANOVA.

(e) Quantification of abundance of LRP1 by fluorescence intensity analysis in WT and 5XFAD mice. N=5 mice per group; Mean ± SEM; *p<0.05 by two-way ANOVA.

(f) Representative laser doppler images of local CBF in 6-month-old SED-WT, EXE-WT, SED-5XFAD and EXE-5XFAD.

(g) Quantification of CBF among groups. Mean ± SEM; N=3 mice per group by two-way ANOVA.

**Figure S3. The size distribution and concentration of exosomes isolated from brain and serum of mice.**

(a and b) Representative results (a) and quantified data (b) of nanoparticle tracking analysis demonstrating size distribution and concentration of brain exosomes among groups. Mean ± SEM; N=5 mice per group by two-way ANOVA.

(c and d) Representative results (c) and quantified data (d) of nanoparticle tracking analysis demonstrating size distribution and concentration of serum exosomes among groups. Mean ± SEM; N=5 mice per group by two-way ANOVA.

**Figure S4. Screening of candidate for miRNAs in the brain exosomes of WT and 5XFAD mice**

(a-d) Quantitative RT-qPCR analysis of other 4 candidate for miRNAs in the brain exosomes of WT and 5XFAD mice. Values are 2^-ΔΔt^; Data are normalized to U6; N=3 mice per group; Mean ± SEM.

**Figure S5. miR-532-5p was essential in the protection of BBB.**

(a) Quantified data of PDGFRβ relative expression by grey values analysis in primary cells transfected with a mimic or inhibitor of miR-532-5p. N=3 replicates; Mean ± SEM; ***p<0.001 by one-way ANOVA.

(b) Quantified data of ZO-1 relative expression by grey values analysis in primary cells transfected with a mimic or inhibitor of miR-532-5p. N=3 replicates; Mean ± SEM; *p<0.05, **p<0.01, ***p<0.001 by one-way ANOVA.

(c) Dye successfully entered the ventricle after ICV (top) and a broad distribution of eGFP in the cortex and hippocampus after ICV of AAV- miR-532-5p (bottom). Scan bar: 1mm.

(d) Quantification of PDGFRβ, NG2, ZO-1 and LRP1 relative expression by grey values analysis of immunoblots showing an increased expression after overexpression of miR-532-5p in 5XFAD mice. β-actin was used as a control. N=4 mice per group; Mean ± SEM; **p<0.01, **p<0.01, ***p<0.001 by two-way ANOVA.

(e) Age-dependent changes in miR-532-5p in the brain of 5XFAD mice. N=3 mice per group; Values are 2^-ΔΔt^; Data are normalized to U6; Mean ± SEM; *p<0.05, **p<0.01 by one-way ANOVA.

(f and g) Quantification of age-related changes in PDGFRβ (f) and ZO-1 (g) relative expression in 5XFAD mice by grey values analysis of immunoblots. β-actin was used as a control. N=3 mice per group; Mean ± SEM; **p<0.01, **p<0.01, ***p<0.001 by one-way ANOVA.

(h) Age-dependent changes in miR-532-5p in the brain of WT mice. N=3 mice per group; Values are 2^-ΔΔt^; Data are normalized to U6; Mean ± SEM; **p<0.01, ***p<0.001 by

(i) Representative blots of PDGFRβ and ZO-1 in the brain of WT mice showing an age-dependent changes in proteins expression.

(j and k) Quantification of age-related changes in PDGFRβ (j) and ZO-1 (k) relative expression in WT mice by grey values analysis of immunoblots. β-actin was used as a control. N=3 mice per group; Mean ± SEM; **p<0.01, **p<0.01, ***p<0.001 by one-way ANOVA.

(l and m) Correlation between age-dependent reduction of ZO-1 (l) or PDGFRβ (m) and miR-532-5p in WT mice. r=Pearson’s coefficient. N=3 mice per group.

**Figure S6. The levels of miR-532-5p in different tissues of WT mice.**

(a) Quantitative RT-qPCR analysis of miR-532-5p in four tissues of SED-WT mice expressed as fold change to the kidney. U6 was used as internal control. N=3; Mean ± SEM; **p<0.01, ***p<0.001 by one-way ANOVA.

(b) Quantitative RT-qPCR analysis of miR-532-5p in four tissues from SED-WT and EXE-WT mice. Data were presented as fold change of SED-5XFAD. U6 was used as internal control. N=3; Mean ± SEM; **p<0.01 by one-way ANOVA.

(c) Representative confocal microscopy images showing miR-532-5p (red) colocalization with CD31^+^ endothelial cells (green) and PDGFRβ^+^ pericytes (magenta) in the cortex and hippocampus of 6-month-old 5XFAD mice. Scan bar: 500μm for left images and 50μm for the right.

(d and e) Quantification of the level of miR-532-5p on pericytes and endothelial cells in the cortex (d) and hippocampus (e) of 5XFAD mice. N=4 mice per group; Mean ± SEM; ***p<0.001 by two-way ANOVA.

**Figure S7. *EPHA4* was a target gene of miR-532-5p. Related to Figure 6**

(a) A list of total target genes of miR-532-5p which are extracted by the intersection of the gene lists from three different databases, including starbase, miRDB, and TargetScan.

(b) Representative blot for EphA4 showing siRNA-EPHA4-2 and siRNA-EPHA4-3 knocked down EphA4 with the greatest efficiency.
